# Influence of the type of training task on intermanual transfer effects in upper-limb prosthesis training: A randomized pre-posttest study

**DOI:** 10.1371/journal.pone.0188362

**Published:** 2017-11-30

**Authors:** Sietske Romkema, Raoul M. Bongers, Corry K. van der Sluis

**Affiliations:** 1 University of Groningen, University Medical Center Groningen, Department of Rehabilitation Medicine, Groningen, The Netherlands; 2 University of Groningen, University Medical Center Groningen, Center of Human Movement Sciences, Groningen, The Netherlands; Washington University in Saint Louis School of Medicine, UNITED STATES

## Abstract

Intermanual transfer, the transfer of motor skills from the trained hand to the untrained hand, can be used to train upper limb prosthesis skills. The aim of this study was to determine the relation between the magnitude of the intermanual transfer effect and the type of training task. The used tasks were based on different aspects of prosthetic handling: reaching, grasping, grip-force production and functional tasks. A single-blinded clinical trial, with a pre-posttest design was executed. Seventy-one able-bodied, right-handed participants were randomly assigned to four training and two control groups. The training groups performed a training program with an upper-limb prosthesis simulator. One control group performed a sham training (a dummy training without the prosthesis simulator) and another control group received no training at all. The training groups and sham group trained on five consecutive days. To determine the improvement in skills, a test was administered before, immediately after, and one week after the training. Training was performed with the ‘unaffected’ arm; tests were performed with the ‘affected’ arm, with the latter resembling the amputated limb. In this study half of the participants trained with the dominant hand, while the other half trained with the non-dominant hand. Participants executed four tests that corresponded to the different training tasks. The tests measured the reaching (movement time and symmetry ratio), grasping (opening time, duration of maximum hand opening, and closing time), grip-force production (deviation of asked grip-force) and functional (movement time) performance. Half of the participants were tested with their dominant arm and half of the participants with their non-dominant arm. Intermanual transfer effects were not found for reaching, grasping or functional tasks. However, we did find intermanual transfer effects for grip-force production tasks. Possibly, the study design contributed to the negative results due to the duration of the training sessions and test sessions. The positive results of the grip-force production might be an effect of the specificity of the training, that was totally focused on training grip-force production. When using intermanual transfer training in novice amputees, specific training should be devoted to grip-force.

## Introduction

Intermanual transfer refers to the phenomenon that after training a motor skill on one arm, the other arm will also improve [1–4]. This finding can be used to improve prosthetic training [5–8]. Early in rehabilitation prosthetic users can start training prosthetic skills on the unaffected arm, so that the skills of the affected arm will enhance. In this way, the training can start directly after the amputation, as a result of which the handling and acceptance of the prosthesis could improve [9–11].

In three previous studies [6–8] we demonstrated intermanual transfer effects after a prosthetic training. Able-bodied subjects trained one arm (dominant side for half of the participants, non-dominant side for the other half), representing the “unaffected” arm, with a prosthesis simulator, a prosthesis that can be worn on a sound arm. Subsequently the other arm, representing the “affected” arm, was tested on motor skills also with the prosthesis simulator. Movement times diminished more in the training group than in a control group [6–8], whereas the control of grip-force training showed no transfer effect. Recently, transfer of prosthetic skills between arms was shown for experienced myo-electric prosthesis users [12]. Movement times of myoelectric prosthesis users, using a prosthetic simulator on the unaffected hand, were shorter, and these users had significantly higher Box and Block Test scores and shorter duration of maximum hand opening than age-matched controls. From literature on intermanual transfer it is known that variations in the degree of transfer depend on characteristics of the training program, such as the learning condition (for example using visual feedback or not), duration of the inter-training intervals [13], or the tasks that are used [14,15]. How different task characteristics influence intermanual transfer effects in prosthetic training has not been investigated yet and was therefore the objective of the current study.

To reveal the magnitude of the intermanual transfer effect for different tasks, we first distinguished three aspects of prosthetic handling: 1) the effect of the extra weight and length of the prosthesis, which will affect reaching, 2) the coordination of the grip, that is, the opening and closing of the prosthesis hand, which should affect grasping, and 3) the accuracy of the grip-force produced during grasping objects with the prosthesis hand. In this study we examined the degree of transfer when a training focuses on each of these individual. Besides the selective training programs we also included a training program, in which all the aspects of prosthesis handling are trained. In this training program the trained tasks vary during training, which might lead to faster learning [15]. Because the variation in tasks over time resemble daily life situations, this training program is regarded as a functional training program. The training programs therefore focused on reaching, grasping, grip-force production and functional tasks.

Due to lack of knowledge about aspects of prosthetic use, the three selected aspects were based on studies using able-bodied participants. The extra weight and length of the prosthesis simulator changes the inertia of the arm during reaching movements. Previous studies show intermanual transfer of the adaptations to the changes in inertia [16,17]. In previous studies the transfer of the coordination of grip-movements has been shown in, among others, dexterity skills [15], a Pegboard task [18], and prosthetic handling [5–8]. Likewise, intermanual transfer effect of control of grip-force has previously been found in precision grip lifting [19,20]. Based on these findings we expected to find an intermanual transfer effect on each of the aspects when used in a training program. Furthermore, the functional training group, where all aspects are combined helps in answering the question whether a variation of training tasks may help participants to learn to handle the prosthesis more proficiently than training just one aspect of prosthesis use.

In previous studies we compared a training program group with a control group that did not follow any training [6,7] or that followed a sham training [8]. The sham training consisted of a dummy training without using the prosthesis simulator. For both the no-training and sham training an intermanual transfer effect was found. To examine the difference between the no-training and sham training, we included both in the current study. With this we intended to establish not only whether non-training differed from training, but also whether training needed to be specific or could be non-specific.

The aim of this study was to reveal if four different training programs, comprising reaching tasks, grasping tasks, control of grip-force tasks, and a combination of these tasks, will all result in intermanual transfer effects. We hypothesize that the (specific) training groups show larger intermanual transfer effects than the sham and no-training groups. It is generally assumed that it is hard to learn to control grip-force with a prosthesis [21–23], therefore intermanual transfer of a grip-force production training program is expected to give at most a small transfer effect. A functional training program, where all aspects of prosthesis tasks are included, comprises most task variability and we hypothesize that such a training program will give the most prominent transfer effect.

## Materials and methods

### Participants

**Fig 1:**
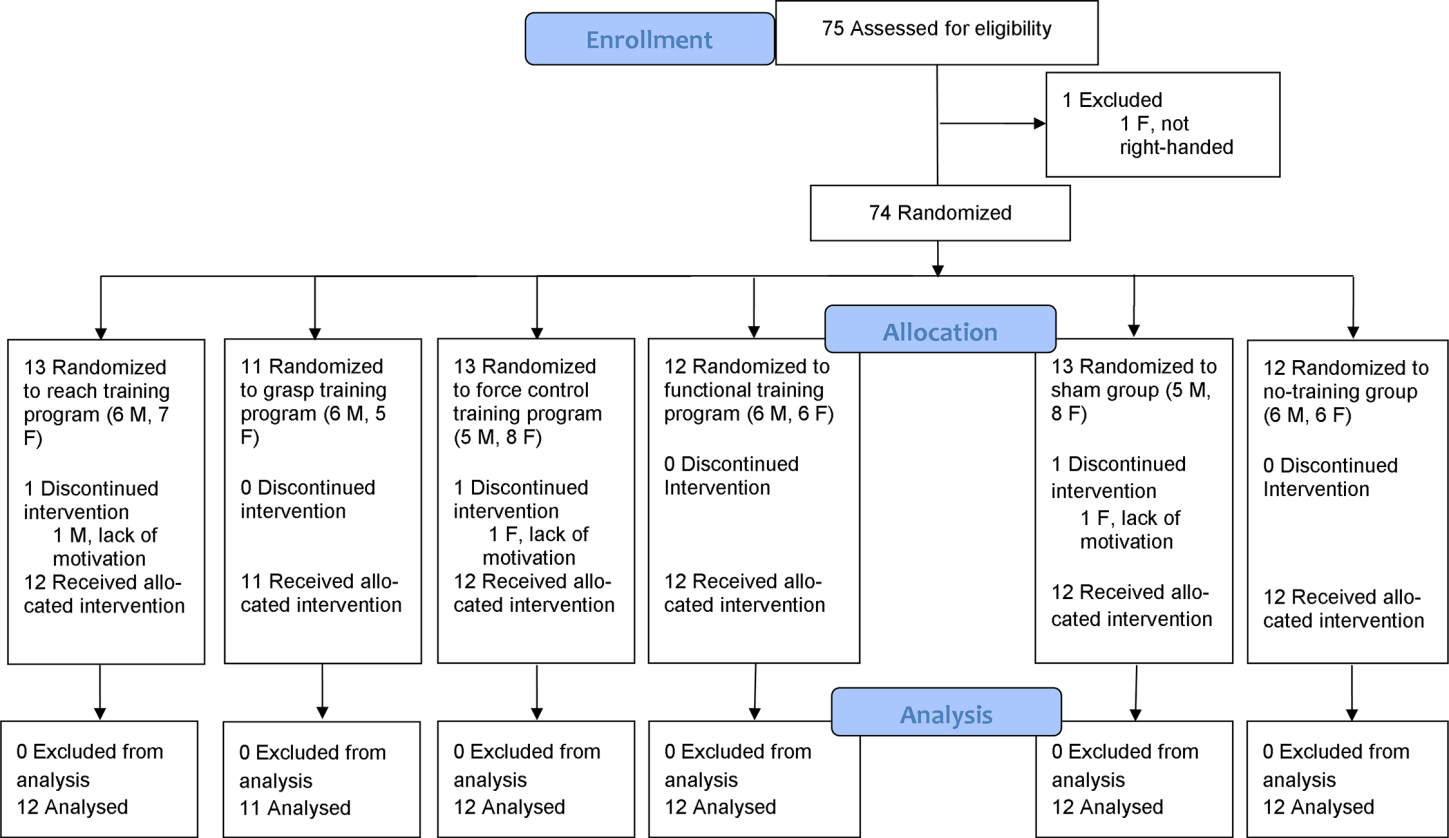
Consort flow diagram.

**Fig 2:**
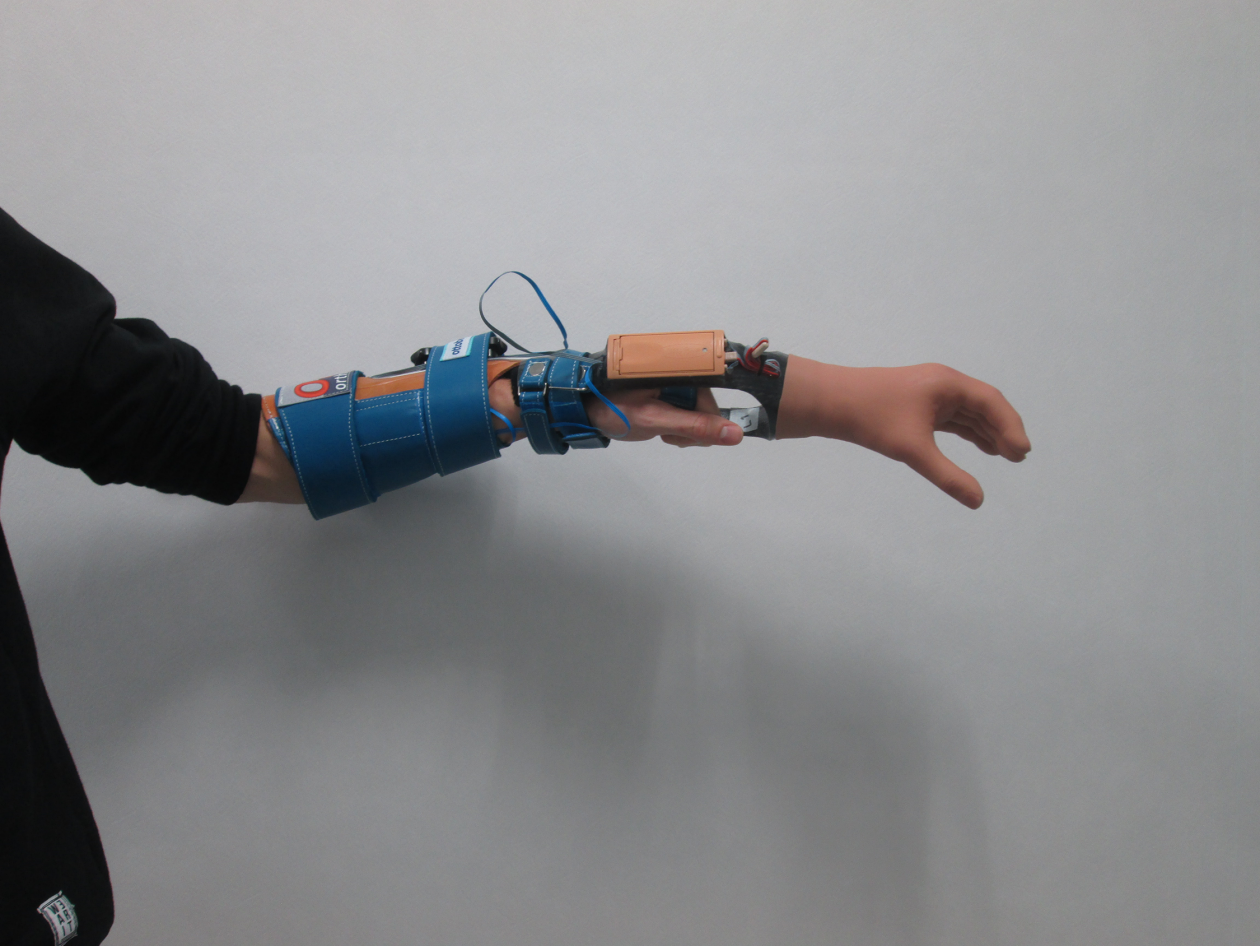
Prosthesis simulator.

Right-handed, able-bodied participants between 18 and 40 years old were recruited and followed at the University of Groningen, the Netherlands, between May 2013 and October 2013 (Fig 1). All participants had normal or corrected to normal sight, were free of neurologic or upper extremity musculoskeletal problems and had no earlier experience with the prosthesis simulator (Fig 2). To assess the handedness we executed the ten item version of the Edinburgh Handedness Questionnaire (EHQ) [24].

The sample size calculation was based on one of the functional tasks of the pretest and posttest of an experiment [6], in which participants also trained five days with the prosthesis simulator. Using G*Power [25], we estimated that we needed ten participants per group to reach a power of 0.8. A t-test with two independent means with an effect size of 0.91 and type I error of 0.05 was used. However, besides measuring improvement within training groups, differences in improvement between training program and control groups was an important concern of the current paper. Since it was expected that these effects might be smaller and controlling for an equal distribution of sex and test hand per group we decided to include 12 participants per group.

### Ethics statement

Before participation all participants signed an informed consent document. The local ethics committee (UMCG Medical Ethics Review Committee, NL43335.042.13) approved the study. The trial was registered with the Nederlands Trial Register (trialregister.nl, NTR3888). After completion of the experiment, participants received a gift voucher.

### Design and randomization

**Fig 3:**
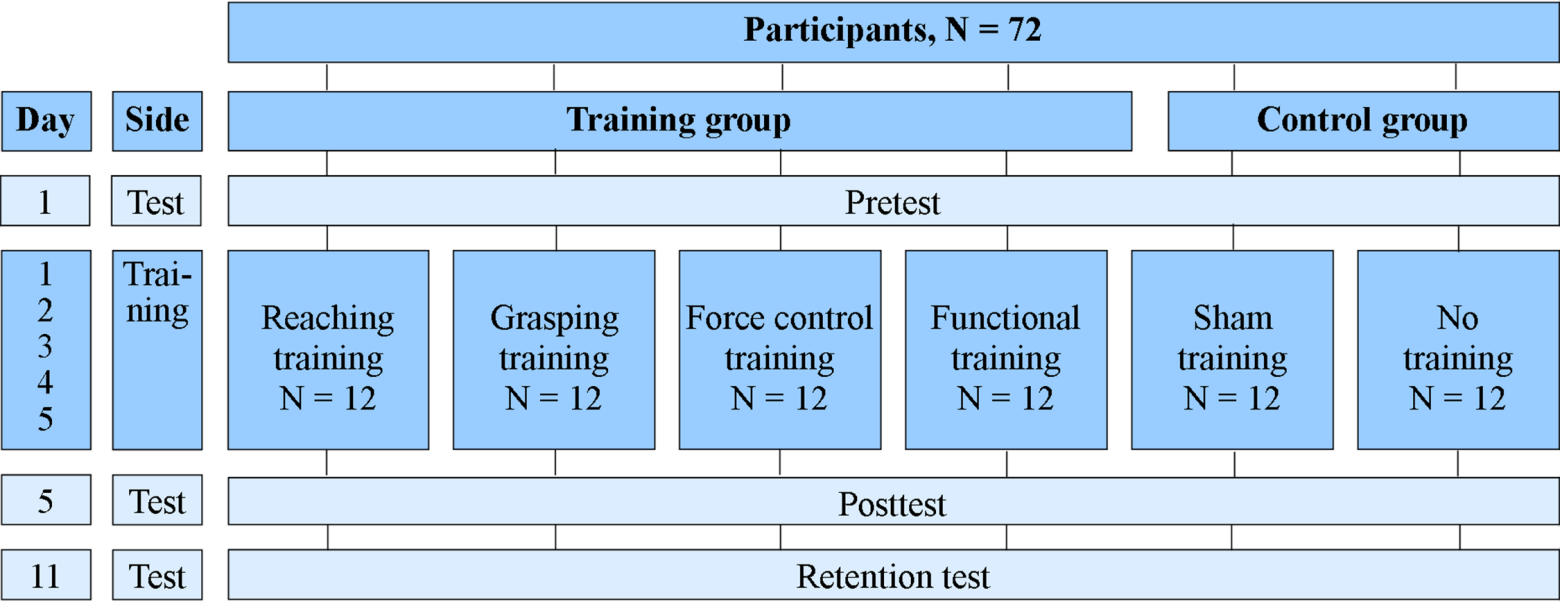
Overview of the design.

Using a computer-generated random number sequence, the participants were randomly assigned to one of six groups (Fig 3); four training program groups (reaching, grasping, grip-force production, and functional) using a prosthetic simulator and two control groups (sham training or no training at all). Each group received a specific training program based on the different aspects of prosthetic handling. All training sessions were executed during 15 minutes and the training tasks in these sessions were executed in a randomized order. The sham group received a sham training, consisting of functional tasks executed with the anatomical hand without the prosthetic simulator. The no-training group did not receive a training at all.

All participants started with the same pretest (day 1) to establish the baseline skills of the participants’ test arm (i.e., affected arm) using a prosthetic simulator. Then, the training groups and the sham group practiced for five days with the opposite (training) arm (day 1–5) (i.e., unaffected arm). Subsequently, all participants performed a posttest (day 5) and a retention test (day 11) using the simulator on the test arm.

The pretest, posttest en retention test consisted of four different test tasks presented in random order. All four test tasks were executed by all participants so that the training and control groups could be compared to each other. Similar to the training programs, each test task contained one of the aspects of prosthetic use. For half of the participants in each group the dominant side was trained and the non-dominant side was tested. For the other half, the non-dominant side was trained and the dominant hand was tested.

### Materials

#### Simulator

The myoelectric prosthesis simulator (OIM Orthopedie, Haren, The Netherlands) [26,27] consisted of an open cast with a myoelectric hand, the MyoHand VariPlus Speed (Otto Bock, Duderstadt, Germany) distally attached to it (Fig 2). The prosthetic hand had proportional speed control (15–300 mm/s) and proportional grip-force production (0-±100 N). The cast extended into a splint along the forearm that could be attached using a Velcro sleeve. The prosthetic hand was controlled by changes in electrical activity related to muscle contraction. The muscle contraction was detected by two electrodes that were placed on the muscle bellies of the wrist extensors and flexors.

#### OPTOTRAK

With the OPTOTRAK 3020 system (Northern Digital, Waterloo, Canada) the movements of the digits of the prosthesis hand in the reaching and grasping tests were recorded. Two infrared light emitting diodes (LED’s) were placed on the top of the ulnar side of thumb and radial side of the index finger of the prosthesis hand. The positions of the two LED’s were recorded from two sides above the table and were sampled with a frequency of 100 Hz. High frequency noise was removed from the data using a second order recursive Butterworth filter with a cut-off frequency of 10 Hz. The data was differentiated once to calculate the velocity and once again for the acceleration using a three point difference algorithm.

### Procedure

All test and training sessions with the prosthesis simulator started with a standard procedure to fit the simulator. The electrodes were placed on the wrist extensor and flexor muscles. The maximum speed of the hand was set to the default setting of six (double channel control, fast open and slower closing). To determine the location of the electrodes and adjust the sensitivity, a MyoBoy (Myoboy; MyoBock Electrodes; Otto Bock, Duderstadt, Germany) was used. To set the sensitivity of the electrodes, the amplified signal had to be hold above a threshold of 1.5 V (high signal) for two seconds. After the simulator was fitted, the participant was positioned in front of the table with the elbow 90 degrees flexed. The participants’ position in front of the table depended on where, during the whole task, the prosthesis hand needed to grasp or hold an object. Verbal instruction on the execution of the tasks was given.

As described in the introduction we aimed to investigate three different aspects of prosthetic handling: 1) the effects of the weight of a prosthesis on reaching, 2) the coordination of opening and closing the prosthetic hand and 3) the production of the grip-force. The logic of the experiment was that the specific aspect was trained while at the same time the other aspects were restricted or kept similar as much as possible. To increase the effect of the training for the specific aspect that was trained, we systematically varied this aspect. For example, when training reaching, the hand opening and closing was turned off. Moreover, to enlarge the learning effects, the weight of the hand affecting inertia, and thus reaching control, was varied in the training sessions. Importantly, during testing both the other aspects were not restricted, and only one condition of the aspect was tested (i.e., no variation of the to be tested aspect was applied during the test).

### Training and test sessions

Each training group had its own training program. The test sessions for all participants consisted of four test tasks all containing one of the training aspects (reaching, grasping, grip-force production and functional). Below, the training tasks, test tasks and specific materials are described for each of the training groups.

#### Reaching training program

**Fig 4:**
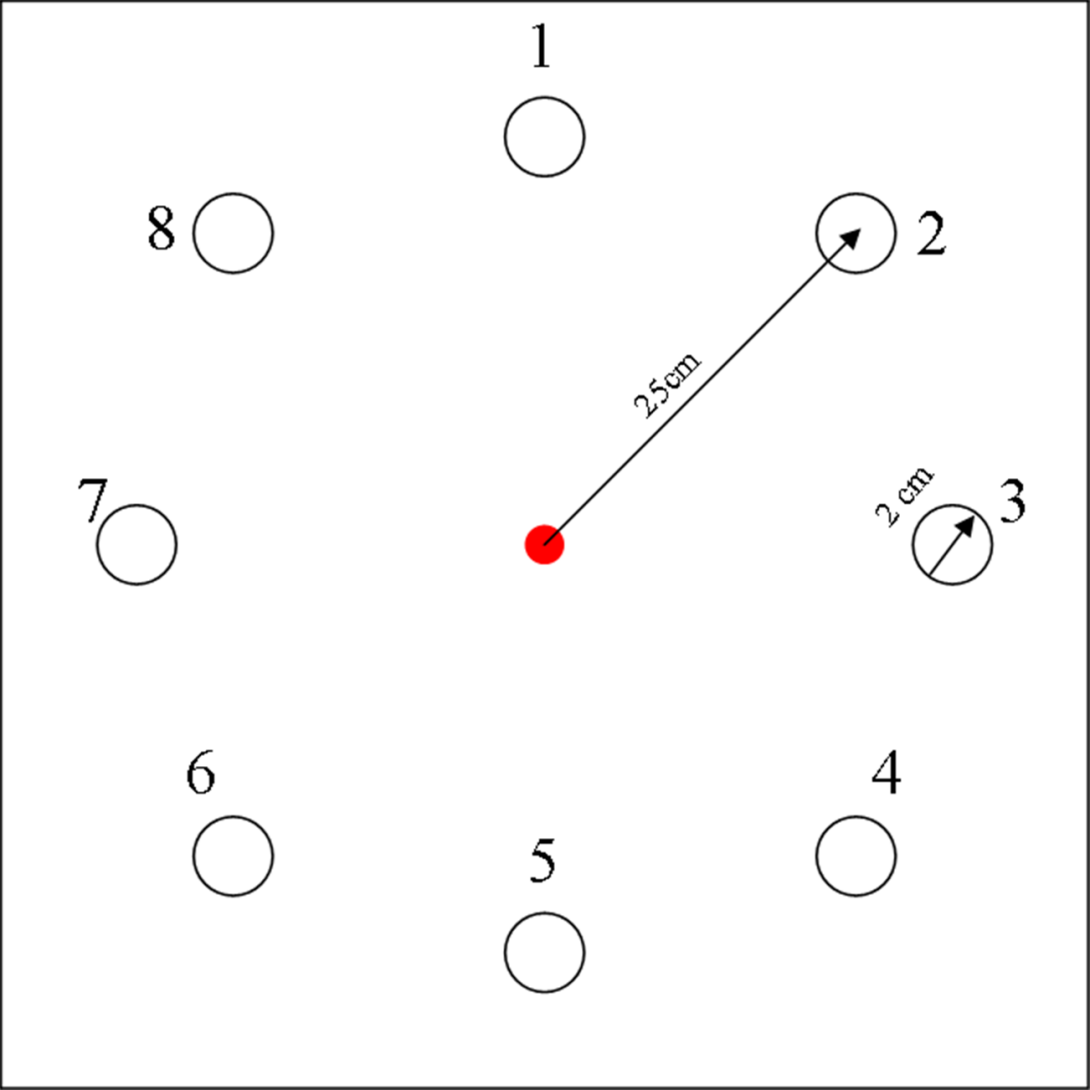
Sheet with reaching goals used during training.

Participants had to reach for targets that were printed on a laminated sheet (51 by 51 cm, Fig 4) laying at a fixed position on the table. They had to reach from the starting position in the middle to different targets numbered 1 till 8. The targets, circles with a diameter of two cm, were all positioned 25 cm from the starting position. This results in an index of difficulty (ID) of 4.6. (computed as ID = log_2_ (2*target distance/target size; Fitts, 1954)).

An erasable marker was placed in the prosthetic hand after which the hand was turned off by the tester. The participant positioned the chair in such a way that all targets could be reached. First, the researcher called a number and after a verbal starting signal, the participant had to reach to the target and put a dot in this target using the marker. The dot gave the participant visual feedback about the result. Participants were asked to perform the movement as fast as possible to make sure that the movement was made fluently.

Using a prosthesis affects the weight of the arm, which affects the forces and torques in the muscles and joints that affect reaching control. To train the handling of the prosthesis three different conditions were applied in which the weight on the arm varied. The weight conditions were: the prosthetic simulator without extra weight, with 500 and with 1000 grams extra weight, respectively. The extra weight was placed around the wrist using Velcro. After eight trials (in all directions) the participant had a short rest. Half way the participant had a rest of two minutes.

#### Reaching test

During the reaching test, participants had to reach to four of the eight targets (1, 3, 5, and 7) to which they reached in the training. Each target had to be reached three times with the simulator holding the marker, without extra weight.

The reaching movements were recorded using OPTOTRAK (see materials). Two variables regarding the reaching movements were derived and analyzed. 1) The movement time, the time used to execute the movement from the starting position till the target. 2) The symmetry ratio of the velocity profile, measured by the acceleration time divided by the movement time [28].

#### Grasping training program

**Fig 5:**
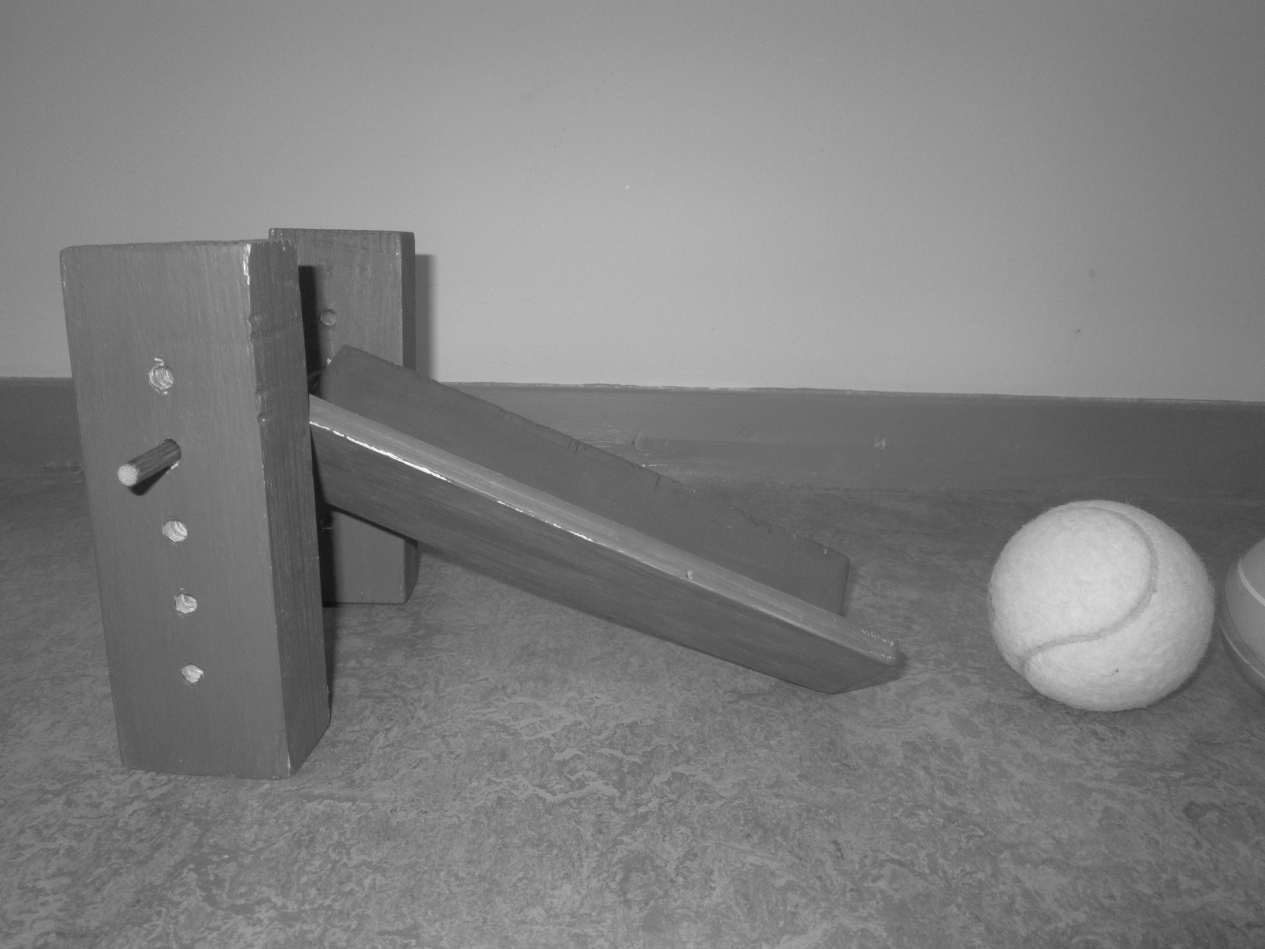
Ramp.

Grasping was trained by letting participants catch balls that were rolling off a ramp with three different slopes (20, 26 and 32 degrees, Fig 5). Five balls with different diameters (40, 45, 50, 65 and 70 mm) were used. Variation was added to the experiment by using different slopes and different ball sizes. The participants started with the prosthetic hand closed. The researcher held the ball at the top of the ramp and let it go after verbally indicating that this was going to happen. Participants were encouraged to catch the ball between the fingertips of the prosthetic hand.

Since we were only interested in the grasping component of this task, the hand of the prosthesis simulator was stabilized with a wooden board with Velcro straps. In this way there was no reaching of the hand. The hand was fixed at 25 cm distance from the ramp, in such a position that the hand only had to be opened en closed to catch the ball. In this way handling the weight, or moment of inertia of the prosthesis, was not trained.

#### Grasping test

During the grasping tests, a ball rolling off the ramp needed to be caught with the prosthetic simulator hand, which was not fixed (i.e., reaching component was involved and thus inertia of the hand could affect movement). Participants held their hand behind a line, which was drawn 30 cm from the ramp. The grasping test was executed three times with the ramp at 20 degrees using a 70 mm ball.

OPTOTRAK was used to measure three variables during grasping: 1) The duration of the hand opening; the time it takes to open the hand, 2) the duration of maximum hand opening in the grasping profile, which was defined as the time from the end of hand opening to the start of hand closure (determined by a threshold of 3 cm/s), and 3) the duration of the hand closing; the time it took to close the hand.

#### Grip-force production training program

To train the control of grip-force delivered with the prosthetic hand, three tasks were used: the compressible object, the tracking and the matching task.

**Fig 6:**
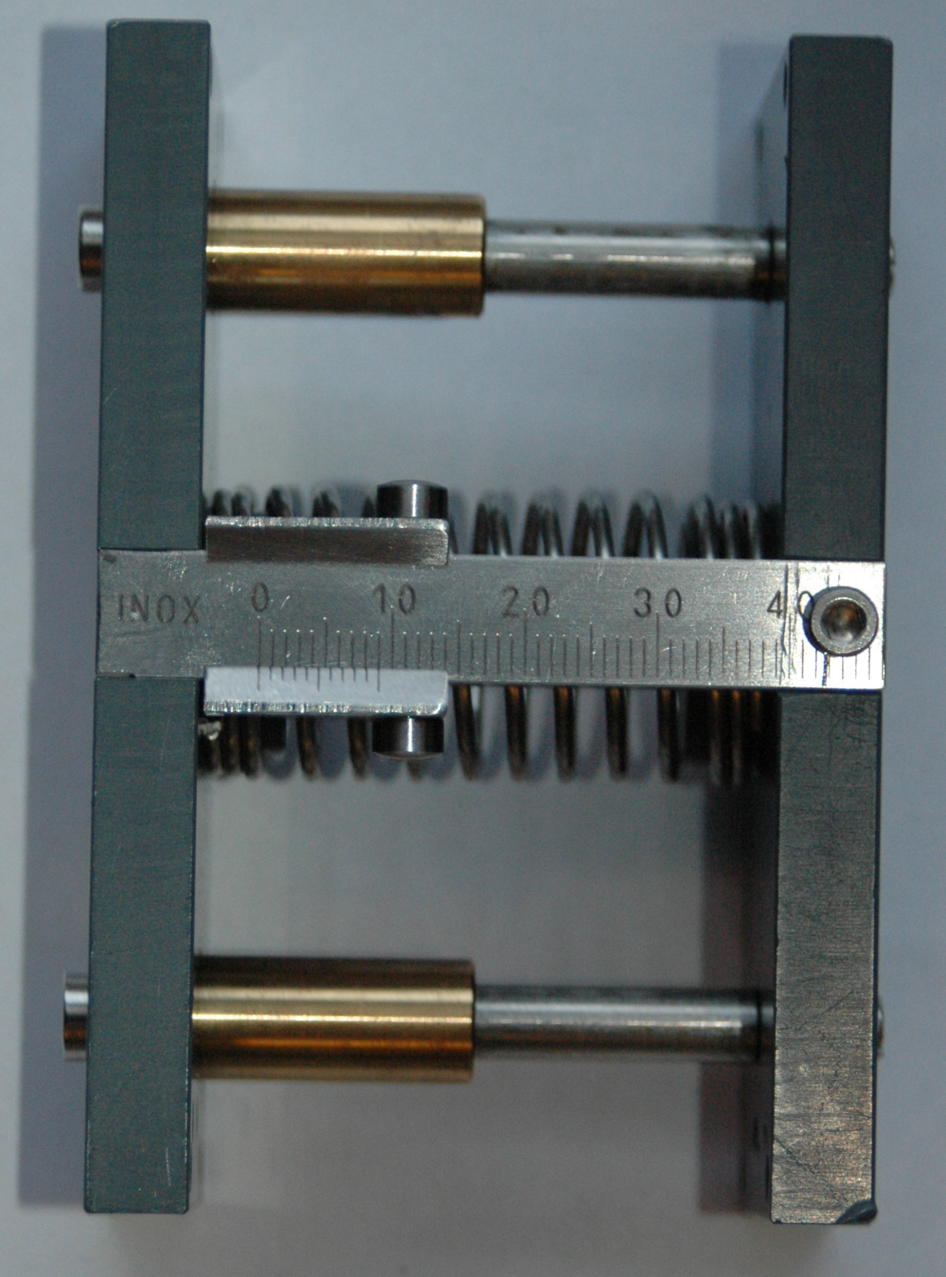
Deformable object.

In the compressible object tasks, three deformable objects were used to train grip-force production [22]. The deformable objects consisted of two plates (6 cm x 3.5 cm x 9 cm) with a spring in between (Fig 6). The springs had a constant of 5.31 N/mm, 0.57 N/mm and 0.17 N/mm. The deformable objects [6] had to be picked up and put on a shelf 25 cm above the table. Participants were instructed to compress the objects as little as possible.

**Fig 7:**
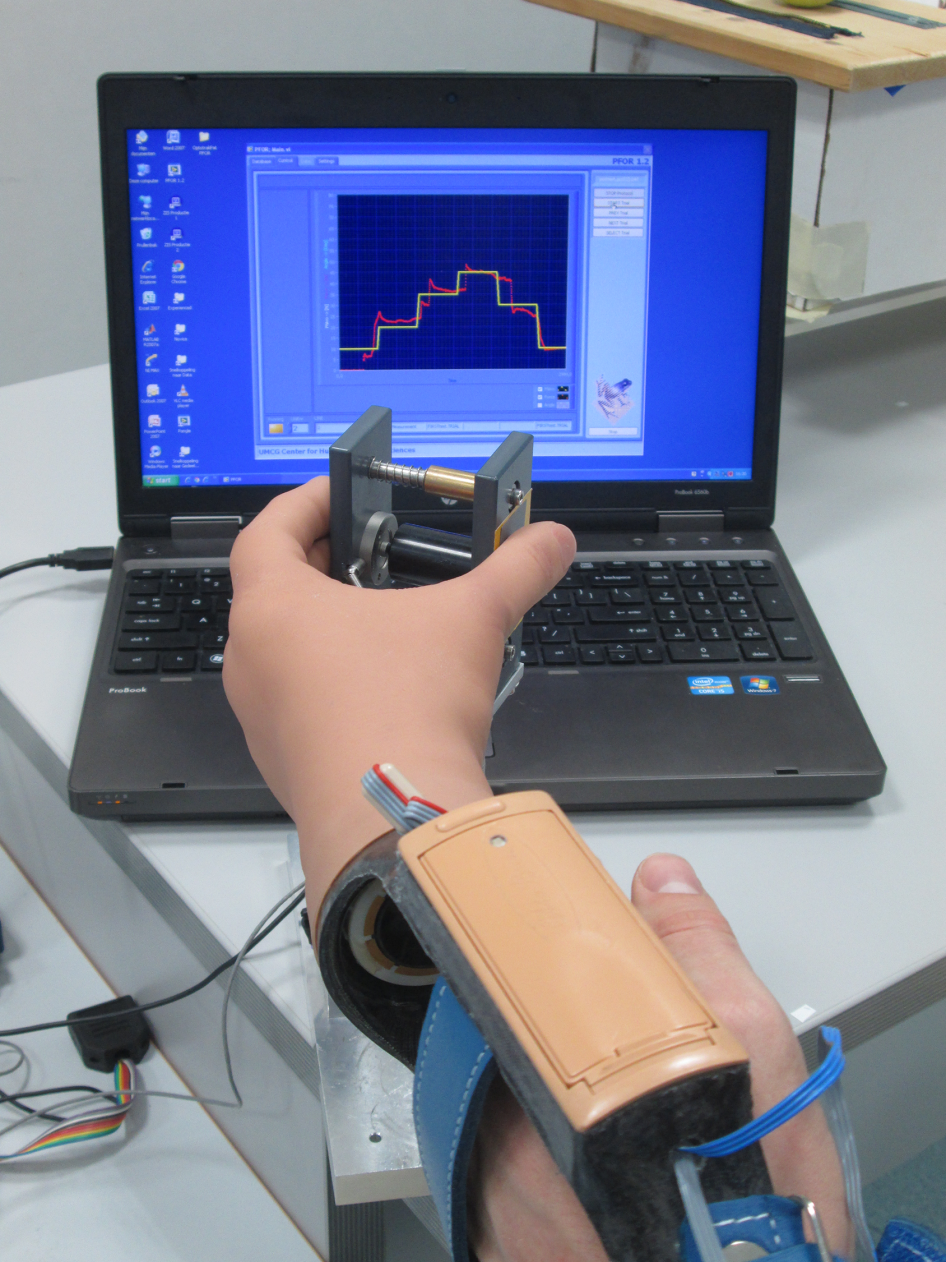
Custom made program measuring the delivered grip-force on the handle during the tracking task.

The tracking and matching tasks were executed using a custom-made computer program, with which the amount of grip-force, when pinching a handle, could be measured. The handle consisted of the same object as a deformable object but had a load sensor placed where the spring was placed in the compressible object and the slider was removed (Fig 7). Two different tasks were used. In the tracking task a pattern on a computer screen needed to be followed for 30 seconds by pressing the handle with the prosthetic hand. By pressing harder the line went up and by loosening grip, the line went down. The requested and performed forces were shown on the screen. The asked pattern consisted of different levels of absolute forces (ranged 5–45 N) that varied in a blocked, sine wave or compound sine wave pattern. The course of the pattern appeared 200 ms before the participant had to produce the grip-force. The pattern started with a line of three seconds at a grip-force of ten Newton, to make sure that participants were able to position the prosthetic hand on the handle, and that all participants had the same starting position. After these first three seconds the blocked pattern started. After each trial the participant was allowed to take a break for a few seconds. For the matching task a handle was squeezed as fast as possible until the amount of grip-force (5–45 N) shown with a cursor on the screen was reached. Both the requested and performed grip-force were shown. The grip-force needed to be hold for ten seconds. After each ten trials the participant was allowed to take a short break.

#### Grip-force production test

To test the improvement in grip-force production a tracking task was performed. Participants executed a blocked pattern three times. The mean absolute deviation of the requested grip-force in N was used as the dependent variable.

#### Functional training program

The functional training tasks consisted of a combination of reaching, grasping and grip-force production and contained ten functional tasks. Part of the tasks (1–7) were derived from the Southampton Hand Assessment Procedure (SHAP; [29]). The remaining tasks were added to specifically include the grip-force production aspect.

1. picking up a light object using the power grip
2. picking up a light tripod object
3. picking up a heavy spherical object
4. pouring water from a carton
5. opening a jar lid using the prosthesis hand
6. picking up four coins
7. cutting clay with a knife
8. picking up a plastic cup filled with water and put it on a 25 cm high shelf
9. picking up an empty plastic cup and put it on a 25 cm high shelf
10. picking up a deformable object and put it on a 25 cm high shelf

#### Functional test

The functional test tasks were based on the preferred use of the prosthesis in daily life [30]; direct grasping, indirect grasping and fixating. For the ‘mug task’ (direct grasping), the participant had to pick up a mug by the handle using the simulator and place it 25 cm above the table on a shelf [27]. In the ‘jar lid task’ (indirect grasping), a jar was picked up by the sound hand and had to be passed to the prosthetic hand, the lid then had to be removed by turning it with the sound hand [27]. In the ‘pen case task’ (fixating), a pencil case was held with the prosthetic hand while the zipper was opened with the sound hand [27]. Before each trial, a computer screen on the left side of the participant showed which task had to be executed. E-Prime (Psychology Software Distribution, Stittenham, York, UK) was used to register the movement time (milliseconds). A keyboard was positioned on the side of the arm that was tested. Participants were instructed to execute all tasks as rapidly and accurately as possible. The movement time was assessed by pressing the space bar before the start and after completion of the task.

#### Sham training program

The sham group executed a training with the sound training hand, without using the prosthetic simulator. During the training the SHAP [29] and Purdue Pegboard dexterity test [31] were performed. The SHAP consists of 26 tasks, twelve abstract tasks and 14 tasks of daily life. The Purdue Pegboard is a board where as many pins should be put in holes during 30 seconds or additionally put collars and washers on it within a minute. This instrument is originally used to measure fine and gross motor dexterity and coordination.

#### No training program

The no-training group did not receive any training. The sham group and the no-training group performed the same four tests as the training program groups.

### Statistical analysis

Analyses were performed using Social Package Statistical Science (SPSS) 22.0 software package (SPSS, IBM Corp in Armonk, NY). The means for all trials in each test were calculated for all the dependent variables (see Table 1). In all analyses, the test results of a specific training program group were compared to the corresponding test results of both control groups. To compare the different tasks of the reaching and the functional tests z-scores were calculated. All outliers that deviated more than three times the standard deviation per test were removed. Missing values were replaced using the expectation maximization algorithm in SPSS.

**Table 1:** Summary of dependent variables for each test.

Test	Dependent variables	Explanation
Reaching	Movement time	The time used to execute the movement from the starting position till the target.
	Symmetry ratio	The acceleration time divided by the movement time.
Grasping	Opening time	The time it takes to open the hand.
	Duration of maximum hand opening	The time from the end of hand opening to the start of hand closure.
	Closing time	The time it took to close the hand.
Grip-force production	Deviation	The mean absolute deviation of the requested grip-force.
Functional	Movement time (z-score)	The time from the release of the space bar and pressing the space bar after completing the task.

Seven repeated-measures ANCOVA’s were conducted, each on one of the depending variables with test (posttest and retention test) as within-subject factor and training program (training program, sham and control) and test hand (dominant or not-dominant) as between-subject factors. Tasks were added as a within-subject factor for the functional (mug, jar-lid and pen case task) and reaching tests (direction 1, 2, 3 and 4). The tasks of the pretest were used as a covariate. Each training group was analyzed separately according to the above described method.

The reason that the pretest was added as a covariate was that the starting level of the different groups, e.g. the possible baseline differences, should not influence the results after the training. Effects of the covariates showed whether the results of the tasks in the pretest affected the findings. To find out if the intermanual transfer effect was present we were interested in main effects of training, where we expected that the training group would perform better than the control and sham group. There might have been an interaction effect of training and session when the training group only improved at the retention test.

To examine whether learning was different for the different tasks we performed one additional ANOVA on the difference between the z-scores of the pretest and the posttest with group as the between-subject variable. For the reaching we used the movement time as the variable, for the grasping we used the plateau time as the variable, for the grip-force production we used the deviation, and for the functional tasks we used the movement time as the dependent variables.

Because we used an extended test battery it could be that participants trained during the pretest. To check for this possibility, we looked into a possible learning trend within the pretest. To compare the different test tasks, z-scores for each test variable were calculated. After that, a one-way ANOVA with the overall z-score as the dependent variable and order of tests (1, 2, 3 and 4) as factor was executed.

When sphericity was violated, the degrees of freedom were adjusted using the Greenhouse-Geisser correction. An alpha of .05 was used for all analysis. Post-hoc tests used Bonferroni corrections. The effect sizes of the significant effects were calculated according to the ŋ^2^_p_ and interpreted according to Cohen’s recommendation [32] of 0.02 for a small effect, 0.13 for a medium effect and 0.26 for a large effect.

## Results

Seventy-five participants were eligible for the study, of whom one had to be excluded, because according to the EHQ she was not right-handed. After randomization three participants dropped out because they did not complete all measurement sessions. Finally, 71 participants (33 M, mean age 22.96 [SD 4.05]) participated in six different groups (Fig 1).

### Reaching

#### Movement times

**Fig 8:**
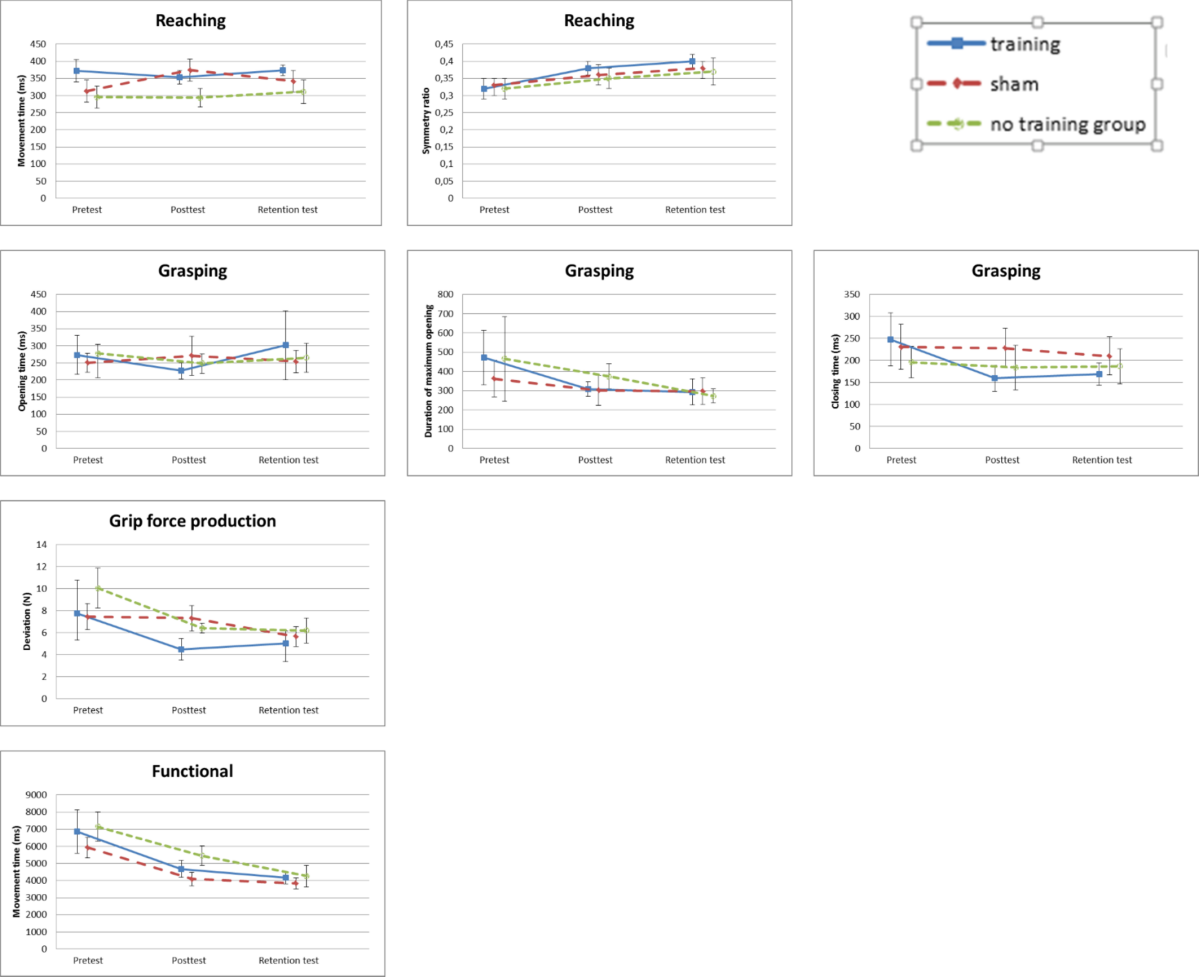
Means (95% confidence interval) for all dependent variables for the groups per test. Note that for the functional tasks the real movement times are shown, while the analyses were performed on the z-scores.

The ANCOVA on showed an effect of training on movement times was found (F_2,14_ = 4.490, P = .031, ŋ^2^_p_ = .391, Fig 8). Post-hoc tests comparing all three training groups while using Bonferroni correction did not show any differences between groups (All P’s = 1.00). Further, an interaction effect of direction and the covariate of reaching in the forward direction (F_3,42_ = 3.180, P = .034, ŋ^2^_p_ = .185) was found. Results for the respective directions differed, but these were as expected and not of primary concern for the present study. Therefore, these effects were not further discussed.

#### Symmetry ratio

A three-way interaction effect for the symmetry ratio of the velocity profile of the reaching movements was found for test, training and test hand (F_2,14_ = 4.533, P = .030, ŋ^2^_p_ = .393, Fig 8). The means showed that the symmetry ratio of the reaching movement was larger for the dominant hand than the non-dominant hand, particularly in the sham group. As a post-hoc test six dependent t-tests were performed examining the difference between the posttest and the retention test. One t-test was executed for both test hands in each group. None of the effects reached the required level of significance after Bonferonni correction. Furthermore, interaction effects of test, training and the right-side direction of the pretest (covariate) (F_2,14_ = 5.476, P = .018, ŋ^2^_p_ = .439) and of test, test hand and the movement in forward direction (F_2,14_ = 7.982, P = .013, ŋ^2^_p_ = .363) were found. Because these interaction effects concern expected differences related to the reaching directions that were not of primary interest in this study these effects were not further discussed.

### Grasping

#### Duration of maximum hand opening

Significant effects in the grasping data were only found for the duration of the maximum hand opening (Fig 8). There was a significant effect of the pretest as covariate (F_1,26_ = 6.689, P = .016, ŋ^2^_p_ = .205) showing that longer duration of maximum hand opening on the pretest resulted in longer duration of the maximum hand opening on the post- and retention test. An interaction effect of training and test hand (F_2,26_ = 5.402, P = .011, ŋ^2^_p_ = .294) was found. To compare both test hands in each of the three training groups, three independent t-tests, using Bonferroni correction were used as post hoc tests. The participants from the sham group using the dominant hand had a shorter duration of the maximum hand opening (t(10) = -1.839, P = .032), while the participants of the control group showed a longer duration of the maximum hand opening using the dominant hand (t(10) = 2.013, P = .024).

### Grip-force production

#### Deviation

A significant effect of training group (F_2,26_ = 7.121, P = .003, ŋ^2^_p_ = .354) was found in the grip-force production data (Fig 8). A post-hoc test, using the Bonferroni correction, where all groups were compared revealed that the grip-force production training group performed better than the sham (P = .002) and the control group (P = .005). As such, for grip-force production the intermanual transfer effect on the untrained arm after training could be demonstrated. Additionally, a significant interaction effect of the training and the pretest as covariate was found (F_2,26_ = 4.486, P = .021, ŋ^2^_p_ = .257). On the pretest the training and sham group had less deviation in grip-force production compared to the control group.

### Functional

#### Movement time

For the movement times of the functional tasks a main effect of one of the covariates was found, namely the jar lid task of the pretest (F_1,18_ = 4.617, P = .046, ŋ^2^_p_ = .204, Fig 8). It was found that the faster the jar-lid task was performed on the pretest, the faster it was performed on the post- and retention tests.

Furthermore an interaction effect of task and training was found (F_4,36_ = 3.046, P = .029, ŋ^2^_p_ = .253). The three training groups were compared for each of the functional tasks in a one-way independent ANOVA. A significant difference was found for the jar-lid task (P = .000) where, using Bonferroni correction, it was found that the control group performed the jar-lid task slower than the training (P = .013) and sham group (P < .001).

### Learning differences between groups

The ANOVA comparing the learning between groups showed no significant effect of groups.

### Test effect

The ANOVA on the possible learning trend within the pretest showed a decreasing trend (F_3,1347_ = 2.950, P = .032, ŋ^2^_G_ = .007) over the order of the tests. However, this trend was very weak.

## Discussion

To determine intermanual transfer effects of different training programs for upper limb prosthesis handling, we compared training programs focusing specifically on reaching, grasping, grip-force production, or functional tasks. We assumed that functional training tasks would lead to relatively large intermanual transfer effects, while grip-force production training would have small transfer effects. Contrary to our expectations, we only found intermanual transfer effects for the grip-force production test. The learning effects in the training did not differ between groups. We found a trend in the improvement over the different tests in the pretest, revealing a learning effect when performing multiple tests, nevertheless this trend was found to be very weak.

This was the first study where we found intermanual transfer effects on the deviation in grip-force production after prosthetic training. Although a transfer effect of grip-force was shown to be possible in precision grip lifting in sound hands [19,20], based on the literature on prosthetic use we expected to find only minimal effects in grip-force production because this aspect of prosthesis handling is hard to learn [21–23]. Nevertheless, this grip-force production training is of importance in everyday life for grasping objects without dropping or breaking them. Interestingly, the change in performance over training in the training arm did not differ between groups, however, only the grip-force production training showed intermanual transfer. An important difference with previous studies that also tested grip-force production [6–8] was that in the current study the training group followed a training that focused solely on the control of force using different types of tasks. The current results seem to suggest that a training program, in which a variety of grip-force production tasks are used, leads to intermanual transfer of the grip-force production.

No significant intermanual transfer effects in the functional test tasks were found, which is different from previous studies on prosthetic training [5–8]. We also did not find transfer effects on the reaching and grasping tasks. Two aspects of the design of our study could have contributed to these results. First, the training sessions were shorter than in our previous studies [6–8]. In these previous studies, where we were able to reveal intermanual transfer effects using a functional training, participants were trained for at least 100 minutes (i.e., five sessions of 20 minutes). Due to the complexity and extent of the current study design this amount of training was not feasible; in the current study participants trained for a total time of 75 minutes. Beforehand, we regarded this training time as appropriate, because this total training time was longer than most other studies on intermanual transfer using a wide range of tasks, performed mostly with the anatomical hand, in which effects of transfer were found [1,4,5,33].

Second, next to the short training sessions, we used extensive test sessions. The duration of the pretests might be a reason that we did not find intermanual transfer effects in the current study. The pretest took more than 15 minutes, during which the participants might already have learned too much about handling the prosthesis with their ‘affected hand’ so that training with the ‘unaffected hand’ did not add anything to this learning. Such a learning effect may not be surprising because it is known from earlier studies [21,34] that the highest learning curve takes place during the first trials. Even more, the test tasks were presented in blocked-random order, the best way to learn prosthetic handling [27]. The results also show learning during the pretest, since, opposite to our previous findings, no further improvement is seen in the posttest and retention test. The lack of improvement over tests might imply that the intermanual transfer effect was too small to add significantly to the learning achieved during the pretest.

A limitation of this study is that the participants were able-bodied subjects. Due to the limited number of persons with an upper-limb amputation it was not feasible to reveal the effects of different training tasks in this target group. Individuals with an amputation will have cerebral reorganization after the amputation, though because intermanual transfer is studied limited in persons with an amputation we do not know how reorganization influences the extent of the transfer effect. Nevertheless a recent study showed that experienced prosthesis users show intermanual transfer effects from the use of the prosthesis on the affected side when using a prosthetic simulator on the unaffected side. Importantly, this study shows that the transfer between the simulator and the actual prosthesis is possible [12].

When applying intermanual transfer in rehabilitation for persons with an upper-limb amputation, according to this study, grip-force production tasks seem the tasks that are most appropriate to use. Previous studies also show transfer effects of functional tasks [5–8]. It needs to be noted that the design of this study might have made the effects of intermanual transfer in the other tasks undetectable. We encourage further research on task specific training because the effects can be important to enable early training after an amputation, with increased performance and acceptance as a result.

## Supporting information

S1 File CONSORT 2010 checklist. (DOC)

S2 File Trial study protocol. (PDF)
