# Supplementary material for: Influence of the type of training task on intermanual transfer effects in upper-limb prosthesis training: A randomized pre-posttest study
Source: PLoS One. 2017 Nov 30;12(11):e0188362. doi: 10.1371/journal.pone.0188362 (PMC5708677; doi:10.1371/journal.pone.0188362)
Supplement: S2 File — (PDF) [file pone.0188362.s002.pdf]

RESEARCH PROTOCOL

Optimizing the intermanual transfer effects after training with a prosthetic simulator

Verwijderd: 1
Verwijderd: 22
Verwijderd: 1

PROTOCOL TITLE 'Optimizing the intermanual transfer effects after training with a prosthetic simulator.'

**Protocol ID**	**43335**
**Short title**	**Optimizing intermanual transfer effects**
**Version**	**1**
**Date**	**22-01-2013**
**Coordinating investigator/project leader**	**Raoul M. Bongers, MSc, PhD<br>Center for Human Movement Sciences, University of Groningen, UMCG, sector F<br>P.O. Box 196<br>9700 AD Groningen<br>(050) 363 8867<br>R.M.Bongers@umcg.nl**<br><br>**Prof. Corry. K. van der Sluis, MD, PhD<br>Center for Rehabilitation<br>UMCG<br>P.O. Box 30.001<br>9700 RB Groningen<br>(050) 361 2295<br>C.K.van.der.Sluis@umcg.nl**
**Principal investigator(s) (in Dutch: hoofdonderzoeker/uitvoerder)**<br>***Multicenter research: per site***	**Sietske Romkema, MSc<br>Centre for Rehabilitation<br>UMCG<br>P.O. Box 30.001<br>9700 RB Groningen<br>(050) 361 0108<br>S.Romkema@umcg.nl**<br><br>**Dr. M Brouwers<br>Revalidatiecentrum de Hoogstraat<br>Rembrandtkade 10<br>3583 TM Utrecht<br>mahbrouwers@gmail.com**
**Sponsor (in Dutch: verrichter/opdrachtgever)**	***C.K.van der Sluis***
**Independent physician**	**Rienk Dekker, MD, PhD<br>Centre for Rehabilitation<br>UMCG<br>P.O. Box 30.001<br>9700 RB Groningen<br>050 – 361 3638<br>R.Dekker@umcg.nl**
**Laboratory sites *<if applicable>***	**Not applicable**
**Pharmacy *<if applicable>***	**Not applicable**

Met opmaak: Engels (V.S.)

Verwijderd: 1
Verwijderd: 22
Verwijderd: 1

PROTOCOL SIGNATURE SHEET

Name	Signature	Date
Sponsor or legal representative:<br>*The research is non-commercial*<br><br>For non-commercial research,<br>Head of Department:<br>Prof. dr. J.H.B Geertzen		8-5-13
Coordinating Investigator/Project leader/Principal Investigator:<br>Prof. Dr. C.K. van der Sluis		8-5-13

Verwijderd: 1
Verwijderd: 22
Verwijderd: 1

TABLE OF CONTENTS

1. INTRODUCTION AND RATIONALE 7
2. OBJECTIVES 9
3. STUDY DESIGN 9
4. STUDY POPULATION 12
   4.1 Population (base) 12
   4.2 Inclusion criteria 12
   4.3 Exclusion criteria 12
   4.4 Sample size calculation 12
5. TREATMENT OF SUBJECTS 13
   5.1 Investigational product/treatment 13
   5.2 Use of co-intervention (if applicable) 13
   5.3 Escape medication (if applicable) 13
6. INVESTIGATIONAL MEDICINAL PRODUCT 13
7. NON-INVESTIGATIONAL PRODUCT 13
8. METHODS 13
   8.1 Study parameters/endpoints 13
      8.1.1 Main study parameter/endpoint 13
      8.1.2 Secondary study parameters/endpoints (if applicable) 14
      8.1.3 Other study parameters (if applicable) 14
   8.2 Randomisation, blinding and treatment allocation 14
   8.3 Study procedures 14
   8.4 Withdrawal of individual subjects 19
      8.4.1 Specific criteria for withdrawal (if applicable) 19
   8.5 Replacement of individual subjects after withdrawal 19
   8.6 Follow-up of subjects withdrawn from treatment 19
   8.7 Premature termination of the study 19
9. SAFETY REPORTING 20
   9.1 Section 10 WMO event 20
   9.2 AEs, SAEs and SUSARs 20
      9.2.1 Adverse events (AEs) 20
      9.2.2 Serious adverse events (SAEs) 20
   9.3 Annual safety report 21
   9.4 Follow-up of adverse events 21
   9.5 Data Safety Monitoring Board (DSMB)/Safety Committee 21
10. STATISTICAL ANALYSIS 21
   10.1 Primary study parameter(s) 21
   10.2 Secondary study parameter(s) 21
   10.3 Other study parameters 21
   10.4 Analysis (if applicable) 21
11. ETHICAL CONSIDERATIONS 21
   11.1 Regulation statement 21
   11.2 Recruitment and consent 21

Met opmaak ... [1]
Gewijzigde veldcode ... [2]
Met opmaak ... [3]
Gewijzigde veldcode ... [4]
Met opmaak ... [5]
Gewijzigde veldcode ... [6]
Met opmaak ... [7]
Gewijzigde veldcode ... [8]
Met opmaak ... [9]
Gewijzigde veldcode ... [10]
Met opmaak ... [11]
Gewijzigde veldcode ... [12]
Met opmaak ... [13]
Gewijzigde veldcode ... [14]
Met opmaak ... [15]
Gewijzigde veldcode ... [16]
Met opmaak ... [17]
Gewijzigde veldcode ... [18]
Met opmaak ... [19]
Gewijzigde veldcode ... [20]
Met opmaak ... [21]
Gewijzigde veldcode ... [22]
Met opmaak ... [23]
Gewijzigde veldcode ... [24]
Met opmaak ... [25]
Gewijzigde veldcode ... [26]
Met opmaak ... [27]
Gewijzigde veldcode ... [28]
Met opmaak ... [29]
Gewijzigde veldcode ... [30]
Met opmaak ... [31]
Gewijzigde veldcode ... [32]
Met opmaak ... [33]
Gewijzigde veldcode ... [34]
Met opmaak ... [35]
Gewijzigde veldcode ... [36]
Met opmaak ... [37]
Gewijzigde veldcode ... [38]
Met opmaak ... [39]
Gewijzigde veldcode ... [40]
Met opmaak ... [41]
Gewijzigde veldcode ... [42]
Met opmaak ... [43]
Gewijzigde veldcode ... [44]
Met opmaak ... [45]
Gewijzigde veldcode ... [46]
Met opmaak ... [47]
Gewijzigde veldcode ... [48]
Met opmaak ... [49]
Gewijzigde veldcode ... [50]
Met opmaak ... [51]
Gewijzigde veldcode ... [52]
Met opmaak ... [53]
Gewijzigde veldcode ... [54]
Met opmaak ... [55]
Gewijzigde veldcode ... [56]
Met opmaak ... [57]
Gewijzigde veldcode ... [58]
Met opmaak ... [59]
Gewijzigde veldcode ... [60]
Met opmaak ... [61]
Gewijzigde veldcode ... [62]
Met opmaak ... [63]
Gewijzigde veldcode ... [64]

11.3 Objection by minors or incapacitated subjects (if applicable) 22
11.4 Benefits and risks assessment, group relatedness 22
11.5 Compensation for injury 22
11.6 Incentives (if applicable) 22
12. ADMINISTRATIVE ASPECTS, MONITORING AND PUBLICATION 22
12.1 Handling and storage of data and documents 22
12.2 Monitoring and Quality Assurance 22
12.3 Amendments 22
12.4 Annual progress report 23
12.5 End of study report 23
12.6 Public disclosure and publication policy 23
13. STRUCTURED RISK ANALYSIS 23
13.1 Potential issues of concern 23
13.2 Synthesis 23

Met opmaak: Engels (V.S.)
Gewijzigde veldcode
Met opmaak: Engels (V.S.)
Gewijzigde veldcode
Met opmaak: Engels (V.S.)
Gewijzigde veldcode
Met opmaak: Engels (V.S.)
Gewijzigde veldcode
Met opmaak: Engels (V.S.)
Gewijzigde veldcode
Met opmaak: Engels (V.S.)
Gewijzigde veldcode
Met opmaak: Engels (V.S.)
Met opmaak: Engels (V.S.)
Gewijzigde veldcode
Met opmaak: Engels (V.S.)
Gewijzigde veldcode
Met opmaak: Engels (V.S.)
Gewijzigde veldcode
Met opmaak: Engels (V.S.)
Gewijzigde veldcode
Met opmaak: Engels (V.S.)
Gewijzigde veldcode
Met opmaak: Engels (V.S.)
Gewijzigde veldcode
Met opmaak: Engels (V.S.)
Gewijzigde veldcode
Met opmaak: Engels (V.S.)
Gewijzigde veldcode

Verwijderd: 1. INTRODUCTION AND RATIONALE . 7¶
2. OBJECTIVES . 9¶
3. STUDY DESIGN . 9¶
4. STUDY POPULATION . 12¶
4.1 Population (base) . 12¶
4.2 Inclusion criteria . 12¶
4.3 Exclusion criteria . 12¶
4.4 Sample size calculation . 12¶
5. TREATMENT OF SUBJECTS . 12¶
5.1 Investigational product/treatment . 12¶
5.2 Use of co-intervention (if applicable) . 12¶
5.3 Escape medication (if applicable) . 12¶
6. INVESTIGATIONAL MEDICINAL PRODUCT . 12¶
7. NON-INVESTIGATI ... [70]

Met opmaak: Engels (V.S.)
Verwijderd: 1
Verwijderd: 22
Verwijderd: 1

LIST OF ABBREVIATIONS AND RELEVANT DEFINITIONS

ABR	ABR form (General Assessment and Registration form) is the application form that is required for submission to the accredited Ethics Committee (ABR = Algemene Beoordeling en Registratie)
AE	Adverse Event
AR	Adverse Reaction
CA	Competent Authority
CCMO	Central Committee on Research Involving Human Subjects
CV	Curriculum Vitae
DSMB	Data Safety Monitoring Board
EU	European Union
EudraCT	European drug regulatory affairs Clinical Trials GCP Good Clinical Practice
IB	Investigator's Brochure
IC	Informed Consent
IMP	Investigational Medicinal Product
IMPD	Investigational Medicinal Product Dossier
METC	Medical research ethics committee (MREC); in Dutch: medisch ethische toetsing commissie (METC)
(S)AE	Serious Adverse Event
SPC	Summary of Product Characteristics (in Dutch: officiële productinfomatie IB1-tekst)
Sponsor	The sponsor is the party that commissions the organisation or performance of the research, for example a pharmaceutical company, academic hospital, scientific organisation or investigator. A party that provides funding for a study but does not commission it is not regarded as the sponsor, but referred to as a subsidising party.
SUSAR	Suspected Unexpected Serious Adverse Reaction
Wbp	Personal Data Protection Act (in Dutch: Wet Bescherming Persoonsgevens)
WMO	Medical Research Involving Human Subjects Act (Wet Medisch-wetenschappelijk Onderzoek met Mensen

Verwijderd: 1
Verwijderd: 22
Verwijderd: 1

SUMMARY

Rationale: To improve the rate of use of prosthetic devices in adults with an upper limb amputation intermanual transfer might be helpful. Intermanual transfer is the ability to transfer motor skills from one, trained side to the other side (Hicks, 1983). This can be used in upper limb amputees by training the unaffected arm while waiting for the prosthesis to be fitted. Especially because it is assumed that training starting early after the amputation will lead to better acceptance and improved prosthetic handling (Malone et al., 1984). Due to intermanual transfer, the prosthetic skills of the affected arm will then improve. Intermanual transfer effects were demonstrated to be present in myo-electric (Romkema, Bongers, & van der Sluis, 2013) and body-powered prosthesis use (Weeks, Wallace, & Anderson, 2003). However, it is unclear how the training program should be like to obtain the largest effects. First, the question rises which tasks the training program should contain to lead to the largest effects of intermanual transfer. Second, it is unknown how the training should be spaced over time for the best results.

Objective: To compare 1) different training tasks and 2) different training intensities to be able to measure which training has the largest effects. And, 3) whether the effects of this training can also be made visible in patients.

Study design: Experiments 1 and 2 are non-blinded randomized trials, experiment 3 is a case series.

Study population: (1) 60 non-amputated adults; (2) 36 non-amputated adults; (3) 4 amputees who will start to use a myo-electric prosthesis for the first time.

Intervention (if applicable): In experiment 1 and 2 in total eight groups of 12 participants train to use a prosthetic simulator for 20 min during 5 days. In experiment 3, four patients with an amputation train (5 times 20 min) with the prosthetic simulator on the unaffected arm. The prosthetic simulator mimics the functioning of a real prosthesis but can be worn by able-bodied participants and at the sound side of an amputee patient. The prosthesis simulator places a prosthetic hand in front of the sound hand.

Main study parameters/endpoints:

- Grip force control: mean deviation of the asked force in N.
- Reaching: mean deviation of the straight path towards the aim in mm
- Grasp: shape of the grasp profile; plateau duration in s.
- Movement time: time taken to execute the movement in s.
- Initiation time: time between the starting signal and the actual start in s.

Nature and extent of the burden and risks associated with participation, benefit and group relatedness:

All participants will use the prosthetic simulator. This simulator mimics a real prosthetic device and can be worn over a sound arm. Because of the use of this simulator we are able to test more participants than only the few recently amputated patients. Importantly, all the measurements are non-invasive and the use of a prosthetic simulator is not different from wearing a regular prosthesis. Therefore, the risks associated with participation can be considered negligible and the burden can be considered minimal.

1. INTRODUCTION AND RATIONALE

To improve the rate of use of prosthetic devices in adults with an upper limb amputation we have been using intermanual transfer. Intermanual transfer implies that when you learn a motor task with one arm, not only that arm improves, but also the arm at the other side becomes better in the specific task (Hicks, Gualtieri, & Schoeder, 1983; Karni et al., 1998; Kumar & Mandal, 2005; Lee, Hinder, Gandevia, & Carroll, 2010; Mier & Petersen, 2006; Pereira, Raja, & Gangavalli, 2011). The untrained side thus benefits from the trained side. In other words, the effect of intermanual transfer is that the prosthetic skills of the affected arm will improve.

The intermanual transfer effect is shown to be present in body-powered (Weeks et al., 2003) and also in myo-electric prostheses (Romkema et al., 2013). With able-bodied participants we showed that after training the 'unaffected' side using a prosthesis simulator, the level of skills at the 'affected' side increased. This effect can be useful in rehabilitation after an upper

Verwijderd: 1

Verwijderd: 22

Verwijderd: 1

limb amputation, because the training can be started earlier. It is assumed that training immediately after the amputation will lead to better acceptance and prosthetic handling (Malone et al., 1984). It is found that training should start within one month after the amputation to achieve maximum success (Atkins, 1992; Dakpa & Heger, 1997; Gaine, Smart, & Bransby-Zachary, 1997). Though in this period often the wounds are not healed yet and the prosthesis is not finished. Using a prosthetic simulator the training at the sound side can start early what might lead to better acceptance and higher prosthesis skills due to effects of intermanual transfer. This might decrease rejection rates of prosthesis devices rejected.

To be able to train the unaffected arm we make use of a prosthetic simulator. With this simulator it is possible to mimic a myo-electric prosthesis. A prosthetic hand can be opened and closed with a motor driven by electrical signals that are produced by muscle activation. The simulator is placed over the arm, and the prosthetic hand is placed before the sound hand (Figure 1) and then operates in the same way as the prosthesis. The training with the simulator is therefore comparable to the training with the myo-electric prosthesis.

In our earlier study (Romkema et al., 2013) where we demonstrated the effect of intermanual training in prosthetic learning we used functional tasks. We found that the movement times increased after a five-day training program. Though, we also showed no significant improvement in the force control, whereas other studies, using the natural hands to grasp an object, had shown intermanual transfer of force control (Bensmail, Sarfeld, Fink, & Nowak, 2010; Buckingham, Ranger, & Goodale, 2012; Chang, Flanagan, & Goodale, 2008; Gordon, Forssberg, & Iwasaki, 1994; Nagel & Rice, 2001). This raises the question of whether we could improve force control through specific training, and how we can obtain the largest intermanual transfer effects of other skills, such as the coordination of reaching and grasping.

To be able to test the prosthetic skills, we determined three aspects of prosthetic skills, namely 1) grip force control, 2) mechanics and 3) grasp coordination. These aspects are chosen because they usually improve after prosthetic training. We will try to reveal how these aspects will improve after different training programs. To be able to examine the effects of different training tasks, participants will be tested in a pre-test post-test, retention test design. In our design, independent of the training program, all participants will perform the tests. In this way we can examine whether training one skill also has an effect on other skills.

Once it is known which tasks should be used for the largest intermanual transfer effects, we also would like to optimize the spacing of the training program. It is generally known that variations in spacing affect the effectiveness on training (Donovan & Radosevich, 1999; Kornmeier & Sosic-Vasic, 2012; Schmidt & Lee, 2005; Shea, Lai, Black, & Park, 2000) For instance, Donovan (1999) found in a meta-analyses of 63 studies, that training in spaced practice conditions leads to significantly higher performance than training in massed practice conditions. Literature on the spacing of training sessions is mostly focused on intertraining periods within 24 hours. Because in this literature the longer intervals (i.e., up to 24 hours) are found to have the largest effects (Goedert & Miller, 2008; Hussain, Sekuler, & Bennett, 2009; Kornmeier & Sosic-Vasic, 2012; Shea et al., 2000; Siengsukon & Boyd, 2009) and because such intertraining time intervals are realistic for rehabilitation we will use intervals of at least 24 hours. It is currently unknown what the effect is of longer time intervals. The current study will examine these longer time intervals because in rehabilitation practice they are often more practical. In an earlier pilot of our group, measuring three people training with the simulator in different intervals, we found the largest effect on the every-day training. This suggests that we might get the largest effect with an every-day training.

When it has been shown how the best results can be obtained, we will test the best design in patients. With this we pretend to study the effects in the main clinical target group; e.g. amputee patients who will obtain a myo-electric prosthesis for the first time. The results of

Verwijderd: 1
Verwijderd: 22
Verwijderd: 1

these patients can be compared to the patients tested with the original design from our earlier study (NL 35268.042.11). The expectation is that novice prosthetic users, who will train with the simulator using the improved program, will develop higher skills in using the prosthesis. The improvement of the training will have consequences for the training program used in rehabilitation.

In conclusion, with this study we aim to optimize the intermanual transfer effects in prosthetic use that we have shown to be present in earlier research. For this we compare different kind of tasks, based on the skills of prosthetic use, and different time intervals between training sessions (i.e., spacing of the training). The first part of this research will be done with able-bodied participants. Including able-bodied participants will mean that we do not have to bother patients who have just been amputated. When the intermanual transfer effect differs due to the change of the training tasks or the length of the intervals, this might have consequences for the training program of patients with traumatic amputations. To be able to find out the consequences for the patients, our target group, we will include four of them practising the most effective training.

2. OBJECTIVES

The objective of our study is to compare 1) different training tasks and 2) different training intensities to be able to measure what training has the largest effects. And, 3) whether the effects of this training can also be made visible in patients.

3. STUDY DESIGN

Three experiments, each with their own design are presented (Table 1-3). In all experiments the same tests are used.

Experiment 1 (Table 1)

The goal of the first experiment is to test with which kind of tasks the intermanual transfer effects are largest in able-bodied adults using the simulator. There will be four experimental groups. The participants in these groups learn to use the simulator on one arm (training arm). The other arm (test arm) is tested to find out if there is an intermanual transfer effect. Each group will train one of the skills necessary to control a prosthesis. The first group trains only force control, e.g. the control over grip force exerted on an object by the prosthesis. The participant is asked not to crush nor drop the objects. The second group trains only the reaching movements to learn to adapt to the changes in inertia caused by the additional weight of the prosthesis. The third group trains reaching movements without the experience of the extra weight of the prosthesis simulator. In this manner the coordination of the grasping (i.e., hand opening and hand closing) is trained. The training program of the last group consists of a combined training of all the three prosthetic skills. The control group (the fifth group) does not receive any training with the prosthetic simulator though is receiving a sham training with their own hand. This group does execute the tests. The different training programs all take 20 minutes and are executed on five consecutive days. In our earlier study we found that transfer effects were clearly visible after five days of training, we expect to be able to measure differences in three days, while still mimicking a realistic rehabilitation setting. The tests consists of a pretest, posttest and retention test (seven days after ending the training), to be able to measure whether there were learning effects and whether these effects remained (see 7.3 for an extensive explanation). All tests consist of the same tasks; functional, grip force control, reaching and grasping tasks. Half of the participants will train their dominant hand and half will train their non-dominant hand.

Table 1. Study design of the first experiment on the training tasks.

Verwijderd: Healthy a[... [71]
Met opmaak: Engels (V.S.)
Verwijderd:
Verwijderd: 1
Verwijderd: 22
Verwijderd: 1

Healthy adults										
Training	**Functional tasks**		**Force control tasks**		**Reaching**		**Grasping**		**Control group**	
Participants	*3 men dom; 3 men n-dom, 3 women dom; 3 women n-dom*		*3 men dom; 3 men n-dom, 3 women dom; 3 women n-dom*		*3 men dom; 3 men n-dom, 3 women dom; 3 women n-dom*		*3 men dom; 3 men n-dom, 3 women dom; 3 women n-dom*		*3 men dom; 3 men n-dom, 3 women dom; 3 women n-dom*	
**Day**	**Test**	**Practice**	**Test**	**Practice**	**Test**	**Practice**	**Test**	**Practice**	**Test**	**Practic**
1	Pretest	20 min	Pretest	20 min	Pretest	20 min	Pretest	20 min	Pretest	-
2	-	20 min	-	20 min	-	20 min	-	20 min	-	-
3	-	20 min	-	20 min	-	20 min	-	20 min	-	-
4	-	20 min	-	20 min	-	20 min	-	20 min	-	-
5	Posttest	20 min	Posttest	20 min	Posttest	20 min	Posttest	20 min	Posttest	-
7	Retention test	-	Retention test	-	Retention test	-	Retention test	-	Retention test	-

Tabel met opmaak

Experiment 2 (Table 2)

The second experiment aims to reveal the effect of spacing on the intermanual transfer. As found in the available literature, for different tasks, the largest effects are found with a period of minimally 24 hours, the longest period that is tested, between training sessions. Though, the topic of spacing has not received a lot of attention in training motor skills and for periods longer than 24 hours. The sparse evidence revealed that the optimal training effects depend on the nature of the task and on the combination of time interval between the training and the time till the retention test. For this study we therefore choose to use three different time intervals with a minimum of 24 hours. The first group trains daily, the second group trains every second day, the third group trains with two and three days intervals between the training sessions. The training tasks will be chosen based on the experiment described in the foregoing; the tasks with the largest effects will be used. Apart from the three tests, as used in the last experiment, there will be an extra retention test. The first retention test takes place on day 17, so that the first retention test is at the same time interval after the first training for all the spacing regimes. The second retention test is conducted two weeks after the last training session. This will make it possible to show the effects of the different intervals till two weeks after the training.

Verwijderd: 0

Table 2. Study design of the second experiment on spacing

Healthy adults						
Spacing	**Short interval**		**Medium interval**		**High interval**	
Participants	*6 men, 6 women (½ dom, ½ n-dom)*		*6 men, 6 women (½ dom, ½ n-dom)*		*6 men, 6 women (½ dom, ½ n-dom)*	
**Day**	**Test**	**Practice**	**Test**	**Practice**	**Test**	**Practice**
1 (Mon)	Pretest	20 min	Pretest	20 min	Pretest	20 min
2 (Tue)		20 min				
3 (Wed)		20 min		20 min		
4 (Thurs)		20 min				20 min
5 (Fri)	Posttest	20 min		20 min		
8 (Mon)				20 min		20 min

Verwijderd: 1
Verwijderd: 22
Verwijderd: 1

9 (Tue)						
10 Wed)			Posttest	20 min		
11 (Thurs)						20 min
12 (Fri)						
15 (Mon					Posttest	20 min
16 (Tue)						
17 (Wed)	Retention test		Retention test		Retention test	
19 (Fri)	Retention test					
24(Wed)			Retention test			
29 (Mon)					Retention test	

.

Experiment 3 (Table 3)
The last experiment is meant to generalize the results to patients with an upper limb amputation. For this experiment patients from Rehabilitation Center De Hoogstraat, Utrecht and the UMCG with an amputation, who will get a myo-electric prosthesis for the first time, will be included. The design of the experiment is comparable to that of the two experiments described above, while the most effective design will be used. After the first experiment is done, two patients will be included that will get a training program based on the most effective training. After the second experiment, another two patients will be included. These patients will get a training program with the most effective training tasks and most effective spacing. The results of these patients will be compared to results of patients who did not receive any additional intermanual training, though did perform the tests. These patients were included in our earlier study. The pretest is left out for all patients, because it is impossible due to the amputation.

Table 3. Study design of the third experiment in patients.

Patients				
Training/spacing	Most effective training, 3 consecutive days.		Most effective training and spacing.	
Participants	2 amputees		2 amputees	
**Session**	**Test**	**Practice**	**Test**	**Practice**
1	-	20 min	-	20 min
2	-	20 min	-	20 min
3	-	20 min	-	20 min
4	-	20 min	-	20 min
5	Posttest	20 min	Posttest	20 min
6	Retention test	-	Retention test	-
7	Retention test	-	Retention test	-

The time of the retention tests for the first two patients is at day 7 and 28. For the second group of patients the retention tests are depending on the findings of the last experiment.

Verwijderd: 1
Verwijderd: 22
Verwijderd: 1

4. STUDY POPULATION

4.1 Population (base)

(1) 60 non-amputated adults; (2) 36 non-amputated adults and (3) 4 patients with an acquired upper limb amputation who will obtain a myo-electric prosthesis for the first time.

4.2 Inclusion criteria

Able-bodied persons

(1) Normal or corrected to normal sight
(2) Right-handed
(3) Aged 18 till 40

Prosthetic users

(1) An unilateral forearm amputation and an indication for a first myo-electric prosthesis

4.3 Exclusion criteria

Able-bodied persons

(1) Neurological problems concerning upper extremity or torso
(2) Motor problems concerning upper extremity or torso
(3) Earlier experience with a prosthetic simulator
(4) Limited sight despite correction

Prosthetic users

(1) An upper limb amputation at a different level than a forearm amputation

4.4 Sample size calculation

To establish the number of adult able-bodied participants that should be included for the first and second experiment we used the data of an earlier study (Romkema et al., 2013). The test tasks in that study are the same as a part of the tasks that we will use in the current study. Based on these data we computed the expected differences in performance of the control group in the pretest and retention test. Using g power, we used the data to estimate the number of participants required to achieve this effect size in our training group. With this procedure we establish that we have to include 9 participants in each training group and the control group to get at a power of 0.8 in our study.

De waarden zijn berekend aan de hand van een pre en posttest van een eerdere trainingsgroep. Deze proefpersonen kregen ook een training gedurende vijf dagen en voerde voor en na deze training deze testen uit.

Tabel 4a

Sample size per group

			power
taaknr	taak	effect size	0.8
1	Mok	1,29	9
2	Etui	1,52	7
3	Jampot	1,78	5
1-3	Alle	1,41	8

Tabel 4b

BEWEGINGSTIJD	group-sessie	
gemiddelde	experimentele	
Taak	pretest	posttest
1	8686	4715
2	7916	5159

Met opmaak: Nederlands (standaard)

Met opmaak: Nederlands (standaard)

Met opmaak: Inspringing: Links: 0 cm

Met opmaak: Inspringing: Links: 0 cm

Tabel met opmaak

Verwijderd: 1

Verwijderd: 22

Verwijderd: 1

3	8589	5236
Eindtotaal	8397	5037

	group-sessie	
stdev	experimentele	
Taak	pretest	posttest
1	4860	1254
2	2866	1965
3	3140	1375
Eindtotaal	3622	1531

Er is gebruik gemaakt van eenzijdige toetsing met een alfa van 0.05.

Met opmaak: Nederlands (standaard)

Met opmaak: Nederlands (standaard)

The used data is from the pre and posttest of an earlier experiment. However in the described experiment we will not only look at the improvement of the experimental group but also at difference in improvement between the experimental and control group. Because the control group is also expected to improve, we assume we need more participants to find a significant effect.
Furthermore, because the data is not normally distributed, we will use more difficult testtasks (especially the force control tasks), and to be able to used equal amounts of men and women and left and right test hands we would like to use 12 participants per group.

Verwijderd: Though

For the third experiment with the patients we are depending on the amount of participants that are available and willing to participate. We need to find participants who recently had an arm amputation and are waiting for a myo-electric prosthesis. We aim to include 4 prosthetic users in order to omit the individual learning curves. Of course this amount of patients is not enough to perform statistical tests. This data is therefore only used as case studies of which descriptive statistics will be presented. With that, it enables us to compare the data with data of an earlier study on patients.

Verwijderd: from

5. TREATMENT OF SUBJECTS

5.1 Investigational product/treatment

The participants learn to use the prosthetic simulator during training sessions. These sessions take place on five days to promote learning. Each training session will take twenty minutes.

Verwijderd: three

5.2 Use of co-intervention (if applicable)

Not applicable

5.3 Escape medication (if applicable)

Not applicable

6. INVESTIGATIONAL MEDICINAL PRODUCT

Not applicable

7. NON-INVESTIGATIONAL PRODUCT

Not applicable

8. METHODS

8.1 Study parameters/endpoints

8.1.1 Main study parameter/endpoint

- Grip force control: mean deviation of the asked force in N is measured in the grip force control tasks.
- Reaching: mean deviation of the straight path towards the target in mm is measured in the reaching task.

Verwijderd: 1

Verwijderd: 22

Verwijderd: 1

- Grasp: length of plateau phase (maximal hand opening) in seconds is measured in the grasping task.
- Movement time: time taken to execute the movement in seconds is measured in the functional task.
- Initiation time: time between the starting signal and the actual start in seconds is measured in the functional task.

8.1.2 Secondary study parameters/endpoints (if applicable)

Not applicable

8.1.3 Other study parameters (if applicable)

Not applicable

8.2 Randomisation, blinding and treatment allocation

The able-bodied adults will be randomly assigned to one of the five (experiment 1) or three (experiment 2) groups (experimental and control group). The number of participants that train with their dominant and non-dominant hand will be equal for both sexes.
The prosthetic users (experiment 3) will be, as far as possible, equally distributed for gender, age and side of amputation over the two groups.

8.3 Study procedures

Prior to the start of the experiment all participants sign an informed consent and it will be explained to them that they can stop with the experiment at any time, without giving a reason.

Design tests and training
To be able to measure improvement we focus on three aspects that can influence prosthetic skill: 1) *grip force control* of the prosthetic hand, 2) *mechanics* and 3) *grasp coordination*. The training programs are all focusing on one of the three skills or a combination of all of them. After executing one of the four training programs the intermanual transfer effect on all skills is measured. With the results it is possible to measure the improvement from different training programs (functional, force control, reaching and grasping) on all different skills.

Table 1. Explanation of the training and measurements of the first experiment

**Training**		**Measurements**		
		GFC	*Mechanics*	*Grasp coordination*
Force control	GFC tasks	x		
Reaching	Aiming towards target		x	
Grasping	Catching a moving ball			x
Functional	Ten functional tasks	x	x	x

x = the training of the aspect of prosthetic skill takes place, transfer is expected
GFC = grip force control

In the experiment the training and test tasks focusing on the same aspects differ from each other. This is to resemble a rehabilitation setting. Not only a single task but the prosthetic skill needs to be learned. In choosing the tasks for the training and test sessions we also take into account the complexity. A relative complex task is used for the training because then the effects on the other arm (of a simpler task) are assumed to be more prominent.

Materials

Verwijderd: 1
Verwijderd: 22
Verwijderd: 1

The myo-electric simulator is developed to closely resemble a myo-electric upper extremity prosthesis for a below-elbow amputation (see Figure 1). The simulator consists of a myo-electric hand, the MyoHand VariPlus Speed® of Otto Bock, attached to an open cast in which the hand can be placed. The cast extends into a splint along the forearm, adjustable in length. The splint can be attached to the arm using a self-adhesive (Velcro) sleeve. The prosthetic hand has proportional speed control (15-399 mm/s) and proportional grip force control (0-±100 N). The hand is controlled by changes in electric muscle activity, detected by 2 electrodes that are placed on the extensors and flexors in the forearm. The exact positions of these electrodes are determined after palpation of the most prominent contraction of muscle bellies of the extensors and flexors. Subsequently, these locations are marked to place the electrodes. The position of the electrodes is then optimized using Otto Bock PAULA®. Hand opening is accomplished by activity of the extensors, while the hand is closed by activity of the flexors. To mimic the use of a prosthesis as closely as possible, the participants are instructed to make minimal movement with the hand, because when one is amputated, the muscles can contract only isometrically.

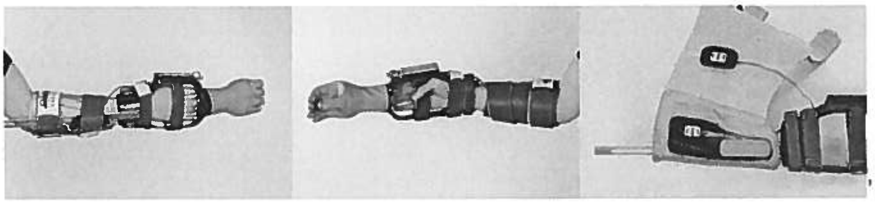

Figure 1 a, b and c. The myo-electric simulator, dorsal, volar side and the electrodes inside.

Otto Bock Paula® (Figure 2) is used in conjunction with 757M11 MyoBoy® with USB connection to a PC for the fitting of the electrodes of the simulator. PAULA stands for Prosthetists' Assistant for Upper Limb Architecture, and is used by prosthetists to evaluate myo-signals and further selection and design of the prosthesis.

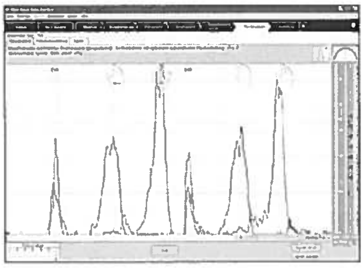

Figure 2. PAULA software on the computer screen, displaying myo-electric signals.

A ramp (Figure 3) is used to let a ball move at the same speed each trial. The ramp is adjustable to different heights. We will use slopes of 10, 15 and 20 degrees.

Verwijderd: 1
Verwijderd: 22
Verwijderd: 1

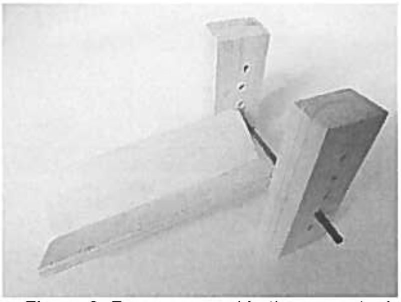

Figure 3. Ramp as used in the grasp tasks.

Two functional tests are used as sham training for the able-bodied subjects. The Southampton Hand Assessment Procedure (SHAP) consisting of 12 abstract and 14 functional tasks and the Purdue Pegboard test for the training hand.

Tests

As described above three aspects of prosthetic performance determine prosthetic skill. The training programs are focusing on one of the three skills or a combination of all of them (functional). The test tasks differ from the training tasks, though they are based on the same skills. The four test tasks (one for each skill) are used to administer the pretests, posttests and one or two retention tests. During the tests the simulator is worn on the test hand (the 'affected' hand) or, in the case of a patient, the prosthesis is used. During training the simulator is worn on the other, 'unaffected' hand. Below, the four test tasks are described in detail.

<u>Grip force control test tasks</u>

Grip force control in this study is the control of the grip force executed on an object that is grasped by the prosthetic hand. Transfer of force control is found to be possible, though it seems to depend on the situation. Maximal force is shown to transfer in different situations, for example (Farthing et al., 2011) grip force control and (Lee & Carroll, 2007) maximal acceleration of finger abduction. Transfer of proportional isometric force is only shown between limbs on two sides of the body, not between upper and lower limbs (Christou & Rodriguez, 2008). Control of force referred towards the environment (Bensmail et al., 2010; Chang et al., 2008; Teixeira, 2000), like in lifting objects, showed better results than force referred towards maximal output, like giving a percentage of the maximal force (Christou & Rodriguez, 2008; Park & Shea, 2002).

In an earlier study we found that after training only functional tasks in prosthetic training, force control of the untrained hand did not improve (Romkema et al., 2013). It is therefore assumed that force control needs to be trained specifically.

We will therefore use a tracking task. In the tracking task a pattern on the screen needs to be followed for 30 seconds by pressing a handle with the prosthetic hand (Figure 4). The pattern consists of different levels of absolute forces (ranged 5 – 45 N) that vary in a blocked pattern. Each amount of force needs to be hold for two seconds. After each trail the participant is allowed to take a break for a few seconds. The course of the pattern appears slightly (200 ms) before the subject has to produce the force. The pattern starts with a line of three seconds of a force of ten Newton, to make sure that participants are able to position the prosthetic hand on the handle, and that all participants have the same starting position. After these first three seconds the blocked pattern starts.

The dependent variable for this test is the mean deviation of the asked force in N.

Verwijderd: 1

Verwijderd: 22

Verwijderd: 1

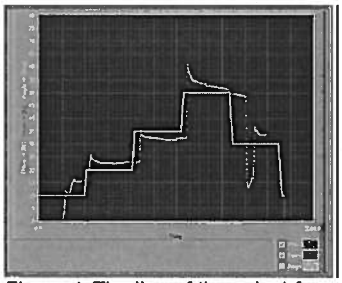

Figure 4. The line of the asked force (yellow) and produced force (red) that can be seen on the computer screen during the tracking task.

Reach test tasks

The participant will reach towards a target 30 cm away while the prosthesis is switched off. The aim is to get the index finger on the target. The target is a circle with a diameter of 3 mm indicated on a table. The shape of the velocity profile and straightness of the trajectory will be analyzed. The dependent variable measured in this test is the mean deviation of the straight path towards the target in mm.

Verwijderd: e

Verwijderd: s

Met opmaak: Tekstkleur: Rood

Grasp test tasks

A ball (60 mm) needs to be caught with the prosthetic simulator. The simulator can freely be moved, without resting on the table. The slope of the ramp is 10 degrees. The ball needs to be caught 30 cm away from the ramp. The dependent variable measured in this test is the length of plateau phase (maximal hand opening) in seconds.

Functional test tasks

The functional tasks consist of three object manipulation tasks, as described by Bouwsema (2008). The tasks are based on the three different ways the prosthesis is handled according to van Lunteren et al. (van Lunteren, van Lunteren-Gerritsen, Stassen, & Zuithoff, 1983); direct grasping, indirect grasping and fixating. In the 'pick-up mug task' the participant has to pick up a mug at the handle with the simulator and to place it 25 cm above the table on a shelf. In the 'lid-off jar task' a jar is picked up by the sound hand at the start and has to be handed over to the simulator, the lid had to be removed by turning it with the sound hand. In the 'zipper task' a pencil case is hold with the simulator at the start position and then the zipper is opened with the sound hand. The dependent variables measured in these tests are the time taken to execute the movement in seconds and the time between the starting signal and the actual start in seconds.

Training

During the training sessions the simulator is worn on the training hand ('unaffected' hand). All training programs are executed during 20 minutes.

Experiment 1

In experiment 1 there are four different tasks for the training programs:

Reach training tasks

While the prosthesis is switched off the participant will reach towards targets. The targets are placed in different directions and consist of circles with a diameter of 3 mm projected on the table. The aim is to reach for the target while the arm has different weights and with that the inertia differs. The weight conditions are: arm with prosthetic simulator, arm with prosthetic simulator and 500 gr extra weight and 1kg extra weight. The extra weight is placed around the arm, just proximal to the wrist.

Verwijderd: 1

Verwijderd: 22

Verwijderd: 1

Grasp training tasks
For this task a grasping movement needs to be made. A ball that rolls towards the participant needs to be caught into the prosthetic hand. The prosthetic simulator is lying on the table, so that the load, and with that the changes in mechanics are not experienced and therefore the participant can not get used to the additional load. The hand is positioned on 30 cm distance from the ramp. Balls of three different sizes are used (40, 50 en 60 mm). The steepness of the ramp is varied (10, 15 and 20 degrees)

Grip force control training tasks
To train the control of force three tasks are used: the tracking, the matching and the object task. The tracking task is similar to the test task. A pattern on the screen is followed by squeezing a handle. The pattern now varies, blocked and sinus patterns are used.
For the matching task a handle is squeezed as fast as possible until the amount of force shown on the screen is reached. The force needs to be hold for ten seconds. The amount of force will be shown with a cursor on a line and differs in a range between 5-45 N. After each ten trails the participant will be allowed to take a short break.
In the object tasks deformable objects (Romkema et al., 2013) are used. The deformable objects have springs with 5 different resistances. Participants have to pick up objects while trying to compress them as minimally as possible.

Functional training tasks
The functional training tasks consist of a combination of the three aspects. It contains of ten functional tasks some of which are coming from the Southhampton hand assessment procedure ((Light, Chappell, & Kyberd, 2002):

1. picking up a light object using the power grip (SHAP)
2. picking up a heavy spherical object (SHAP)
3. picking up a light tripod object (SHAP)
4. pouring water from a carton (SHAP)
5. picking up a full plastic cup
6. picking up an empty plastic cup
7. picking up a deformable object
8. opening a jar lid using the prosthesis hand (SHAP)
9. picking up four coins (SHAP)
10. cutting (SHAP)

Control group training tasks
The control group executes sham training. Here fore we will use the SHAP and Purdue Pegboard dexterity test using the sound hand. Each training session both tests are executed during twenty minutes.

Experiment 2 and 3
The training program that shows the best results will be used for the second experiment. Here only the intensity of the training will be changed over the different groups. The same training is also used for the third experiment on patients.

Procedure
Before the first measurement the Edinburgh Handedness Inventory (Oldfield, 1971) is filled in; only right handed participants are included. This questionnaire consists of 10 Items and it takes 1 minute to fill in.

Before each training and test session, a standard protocol is conducted in order to fit the simulator. The simulator is fitted with help of Otto Bock PAULA®. The electrodes within the simulator have to be placed on the optimal locations. The settings need to be tailored to each individual in order to record the myo-electric signal properly. The

Verwijderd: 1
Verwijderd: 22
Verwijderd: 1

sensitivity of the electrodes will be adjusted for each participant on each day, so that all participants can just reach the myo-electric threshold of 1.5 V (high signal) and hold it for 2 seconds. The maximum speed of the hand is set to the default setting of 6 (range = 1-6). After the simulator is installed, the participant will be seated at a table and the session will start.

Prior to each task, during the training as well as the tests, the experimenter gives the participant instructions to execute the task. The participants are told to sit comfortably at a table, with their arms resting on the table. They start each task with the prosthetic hand closed. The participants are instructed to execute the tasks as rapidly as possible.

Pretest
During all tests we will use the Optotrak system to measure the movements of the arm and prosthesis. Two markers placed on the finger tips of the prosthetic arm; one on the thumb and one on the index finger.
At the first day, the four test tasks are administered to determine the level of skills of the participants of the experimental and control groups of the first, second and third experiment. This test is performed with the test hand.

Training sessions
For the second experiment different intervals between the training sessions are used. The training program in which the spacing will be varied is based on the findings of Experiment 1; the most effective training of Experiment 1 will be chosen to use for the second and third experiment.
Again, the most effective spacing is used in the study on patients.

Posttest and retention test
All participants perform a posttest, equal to the pretest. Four days after the posttest (experiment 1) or on day 10 and two weeks after the posttest (experiment 2) these participants execute a retention test. For both tests the participants again perform the four test tasks, in order to determine the improvement of skills and compare the different groups. The tasks are once more presented in a randomized order and executed with the test hand.

8.4 Withdrawal of individual subjects
Participants can leave the study at any time for any reason if they wish to do so without any consequences. The investigator can decide to withdraw a participant from the study for urgent medical reasons. Since the experiments are safe, we do not expect any urgent medical occasions.

8.4.1 Specific criteria for withdrawal (if applicable)
Not applicable

8.5 Replacement of individual subjects after withdrawal
If a participant withdraws from the study, another participant will be asked to join the study, preferably of the same sex.

8.6 Follow-up of subjects withdrawn from treatment
Not applicable

8.7 Premature termination of the study
Not applicable

Verwijderd: 1
Verwijderd: 22
Verwijderd: 1

9. SAFETY REPORTING

9.1 Section 10 WMO event

In accordance to section 10, subsection 1, of the WMO, the investigator will inform the participants and the reviewing accredited METC if anything occurs, on the basis of which it appears that the disadvantages of participation may be significantly greater than was foreseen in the research proposal. The study will be suspended pending further review by the accredited METC, except insofar as suspension would jeopardise the participants' health. The investigator will take care that all participants are kept informed.

9.2 AEs, SAEs and SUSARs

9.2.1 Adverse events (AEs)

Adverse events are defined as any undesirable experience occurring to a subject during the study, whether or not considered related to the experimental intervention (e.g. prosthetic simulator training or testing). All adverse events reported spontaneously by the subject or observed by the investigator or his staff will be recorded.

9.2.2 Serious adverse events (SAEs)

A serious adverse event is any untoward medical occurrence or effect that at any dose:

- results in death;
- is life threatening (at the time of the event);
- requires hospitalisation or prolongation of existing inpatients' hospitalisation;
- results in persistent or significant disability or incapacity;
- is a congenital anomaly or birth defect;
- Any other important medical event that may not result in death, be life threatening, or require hospitalization, may be considered a serious adverse experience when, based upon appropriate medical judgement, the event may jeopardize the subject or may require an intervention to prevent one of the outcomes listed above.

The investigator is responsible for reporting all SAE's to the sponsor. This is ndependent of the centre where the research takes place. The sponsor will report the SAEs through the web portal *ToetsingOnline* to the accredited METC that approved the protocol, within 15 days after the sponsor has first knowledge of the serious adverse reactions.

SAEs that result in death or are life threatening should be reported expedited. The expedited reporting will occur not later than 7 days after the responsible investigator has first knowledge of the adverse reaction. This is for a preliminary report with another 8 days for completion of the report.

Verwijderd: 1
Verwijderd: 22
Verwijderd: 1

Verwijderd: Not applicable¶

9.3 Annual safety report
Not applicable

9.4 Follow-up of adverse events
Not applicable

9.5 Data Safety Monitoring Board (DSMB)/Safety Committee
Not applicable

10. STATISTICAL ANALYSIS

10.1 Primary study parameter(s)
Deviation of grip force control,
Deviation of straight reaching path,
Length of plateau phase in grasping,
Movement time and initiation time.

10.2 Secondary study parameter(s)
Age
Gender

Verwijderd: Not applicable

10.3 Other study parameters
Not applicable

10.4 Analysis (if applicable)
In all experiments all measurements (deviation of grip force control, deviation of straight reaching path, length of plateau phase in grasping, movement time and initiation time) are subjected to a repeated-measures ANOVA with test (pre-test, post-test and retention test) as within-subject factor and dominance (preferred, non-preferred) and group (experiment 1: 4 experimental groups and control group; experiment 2: three experimental groups) as between-subject factors. In the third experiment, the same effects are analyzed. Though, because there is only a small group this is done by ..... When a Mauchly test indicates that sphericity is violated, the degrees of freedom are adjusted with the Greenhouse-Geisser correction. In all analyses, a significant criterion of α less than or equal to 0.05 is used, and post hoc tests on main effects use Bonferroni adjustment.

Verwijderd: Interim
Verwijderd: a
Verwijderd: A
Met opmaak: Markeren

11. ETHICAL CONSIDERATIONS

11.1 Regulation statement
The study will be conducted according to the principles of the Declaration of Helsinki (59, October 2008) and in accordance with the Medical Research Involving Human Subjects Act.

11.2 Recruitment and consent
The able-bodied participants will be recruited by advertisement on publication boards of different faculties of the University of Groningen, and information presented in course lectures of Human Movement Sciences and Medicine by the investigator. To include the patients, physicians from the Department of Rehabilitation Medicine, UMCG and Rehabilitation Center De Hoogstraat Utrecht are informed about this study and asked to inform patients. Patients will be told that this study is conducted and asked whether a researcher may contact them for information about the study.

Verwijderd: and therapists
Verwijderd:
Verwijderd: recruit
Verwijderd: 1
Verwijderd: 22
Verwijderd: 1

The participants will receive an information letter, with written information about the experiment, after they have shown interest in participating in the experiment. Participants will get between 1 and 8 weeks to decide whether they would like to join the study. For each potential participant there is the possibility to consult the researcher or an independent physician for any further information, this is also mentioned in the letter. After participants have signed in, they will sign an informed consent before the start of the experiment and it will be explained to them that they can stop with the experiment at any time without giving a reason. This can be done by telling the researchers.

Verwijderd: T

Verwijderd: at

11.3 Objection by minors or incapacitated subjects (if applicable)

Not applicable

11.4 Benefits and risks assessment, group relatedness

The participants have to learn to use a simulator during training sessions and will be tested on their abilities. All training sessions are done with non-injured hands and the measurements are non-invasive. Therefore, the risks associated with participation can be considered negligible and the burden can be considered minimal.

11.5 Compensation for injury

Because participation in the experiment is without risks, the judging committee, the METc UMCG has granted a release from compulsory insurance, as referred to in section 4 paragraph 1 of the 'Besluit verplichte verzekering bij medisch-wetenschappelijk onderzoek met mensen'.

11.6 Incentives (if applicable)

Not applicable

12. ADMINISTRATIVE ASPECTS, MONITORING AND PUBLICATION

12.1 Handling and storage of data and documents

The data is handled confidentially and coded for each participant; each participant will be given a number from 1 to 100. The investigator will keep the data for the duration of the project. The handling of personal data is complied with the Dutch Personal Data Protection Act (De Wet Bescherming Persoonsgegevens, Wbp).

Verwijderd: Therefore, all data is anonymous after coding.

12.2 Monitoring and Quality Assurance

Monitoring of the conduct of the study takes place by R.M. Bongers. The aim is to verify the rights and well-being of the participants, to check if the reported information is correctly derived from the original data and if the execution of the experiment in consensus is with the protocol, with good clinical practice and relevant laws. The inclusion of participants, the possible advents, the execution of the study and the progress of the study are monitored. The monitoring takes place at least once a week and in the lab where the experiments are conducted.

Verwijderd: Not applicable¶

Met opmaak: Engels (V.S.)

12.3 Amendments

Amendments are changes made to the research after a favourable opinion by the accredited METC has been given. All amendments will be notified to the METC that gave a favourable opinion.
The amendments will be implemented after the METc gives a positive judgement.

Verwijderd: 1

Verwijderd: 22

Verwijderd: 1

Non-substantial amendments will not be notified to the accredited METC and the competent authority, but will be recorded and filed by the sponsor.

12.4 Annual progress report

The investigator will submit a summary of the progress of the trial to the accredited METC after a year. Information will be provided on the date of inclusion of the first subject, numbers of subjects included and numbers of subjects that have completed the trial, serious adverse events/ serious adverse reactions, other problems and amendments.

12.5 End of study report

The investigator will notify the accredited METC of the end of the study within a period of 8 weeks. The end of the study is defined as the last patient's last visit.
In case the study is ended prematurely, the investigator will notify the accredited METC, including the reasons for the premature termination.
Within one year after the end of the study, the investigator/sponsor will submit a final study report with the results of the study, including any publications/abstracts of the study, to the accredited METC.

12.6 Public disclosure and publication policy

The results of this study will be unreservedly published in a peer reviewed scientific journal.

13. STRUCTURED RISK ANALYSIS

In this study a medical device, e.g. the prosthetic simulator, will be used.

13.1 Potential issues of concern

In this study we will use a medical device, namely the prosthetic simulator. We expect that this study has no risk of potential issues of concern for the participants. The used prosthetic simulator is an approved medical device (approval is added). Furthermore, the device is used before in two experiments of our study group (NL26993.042.09 en NL35268.042.11).

13.2 Synthesis

Not applicable

REFERENCES

References

Atkins, D. (1992). Adult upper limb prosthetic training. In H. K. Bowker, & J. W. Michael (Eds.), *Atlas of limb prosthetics: Surgical, prosthetic, and rehabilitation principles* (2nd ed.,) Rosemont, I.L.

Bensmail, D., Sarfeld, A. S., Fink, G. R., & Nowak, D. A. (2010). Intermanual transfer of sensorimotor memory for grip force when lifting objects: The role of wrist angulation.

Verwijderd: 1
Verwijderd: 22
Verwijderd: 1

Clinical Neurophysiology : Official Journal of the International Federation of Clinical Neurophysiology, 121(3), 402-407. doi: 10.1016/j.clinph.2009.11.010

Buckingham, G., Ranger, N. S., & Goodale, M. A. (2012). Handedness, laterality and the size-weight illusion. *Cortex; a Journal Devoted to the Study of the Nervous System and Behavior, 48*(10), 1342-1350. doi: 10.1016/j.cortex.2011.09.007; 10.1016/j.cortex.2011.09.007

Chang, E. C., Flanagan, J. R., & Goodale, M. A. (2008). The intermanual transfer of anticipatory force control in precision grip lifting is not influenced by the perception of weight. *Experimental Brain Research.Experimentelle Hirnforschung.Experimentation Cerebrale, 185*(2), 319-329. doi: 10.1007/s00221-007-1156-0

Christou, E. A., & Rodriguez, T. M. (2008). Time but not force is transferred between ipsilateral upper and lower limbs. *Journal of Motor Behavior, 40*(3), 186-189. doi: 10.3200/JMBR.40.3.186-189

Dakpa, R., & Heger, H. (1997). Prosthetic management and training of adult upper limb amputees. *Current Orthopaedics, 11*, 193-202.

Donovan, J. J., & Radosevich, D. J. (1999). A meta-analytic review of the distribution of practice effect: Now you see it, now you don't. *Journal of Applied Psychology, 84*(5), 795-805.

Farthing, J. P., Krentz, J. R., Magnus, C. R., Barss, T. S., Lanovaz, J. L., Cummine, J., . . . Borowsky, R. (2011). Changes in functional magnetic resonance imaging cortical activation with cross education to an immobilized limb. *Medicine and Science in Sports and Exercise, 43*(8), 1394-1405. doi: 10.1249/MSS.0b013e318210783c; 10.1249/MSS.0b013e318210783c

Met opmaak: Engels (V.S.)

Verwijderd: 1

Verwijderd: 22

Verwijderd: 1

Gaine, W. J., Smart, C., & Bransby-Zachary, M. (1997). Upper limb traumatic amputees. review of prosthetic use. *Journal of Hand Surgery (Edinburgh, Scotland), 22*(1), 73-76.

Goedert, K. M., & Miller, J. (2008). Spacing practice sessions across days earlier rather than later in training improves performance of a visuomotor skill. *Experimental Brain Research.Experimentelle Hirnforschung.Experimentation Cerebrale, 189*(2), 189-197. doi: 10.1007/s00221-008-1414-9; 10.1007/s00221-008-1414-9

Gordon, A. M., Forssberg, H., & Iwasaki, N. (1994). Formation and lateralization of internal representations underlying motor commands during precision grip. *Neuropsychologia, 32*(5), 555-568.

Hicks, R. E., Gualtieri, C. T., & Schoeder, S. R. (1983). Cognitive and motor components of bilateral transfer. *Amercan Journal of Psychology, 96*(2), 223-228.

Hussain, Z., Sekuler, A. B., & Bennett, P. J. (2009). How much practice is needed to produce perceptual learning? *Vision Research, 49*(21), 2624-2634. doi: 10.1016/j.visres.2009.08.022; 10.1016/j.visres.2009.08.022

Karni, A., Meyer, G., Rey-Hipolito, C., Jezzard, P., Adams, M. M., Turner, R., & Ungerleider, L. G. (1998). The acquisition of skilled motor performance: Fast and slow experience-driven changes in primary motor cortex. *Proceedings of the National Academy of Sciences of the United States of America, 95*(3), 861-868.

Kornmeier, J., & Sosic-Vasic, Z. (2012). Parallels between spacing effects during behavioral and cellular learning. *Frontiers in Human Neuroscience, 6*, 203. doi: 10.3389/fnhum.2012.00203; 10.3389/fnhum.2012.00203

Kumar, S., & Mandal, M. K. (2005). Bilateral transfer of skill in left- and right-handers. *Laterality, 10*(4), 337-344.

Verwijderd: 1
Verwijderd: 22
Verwijderd: 1

Lee, M., & Carroll, T. J. (2007). Cross education: Possible mechanisms for the contralateral effects of unilateral resistance training. *Sports Medicine (Auckland, N.Z.), 37*(1), 1-14.

Lee, M., Hinder, M. R., Gandevia, S. C., & Carroll, T. J. (2010). The ipsilateral motor cortex contributes to cross-limb transfer of performance gains after ballistic motor practice. *Journal of Physiology, 558*(1), 201-212.

Light, C. M., Chappell, P. H., & Kyberd, P. J. (2002). Establishing a standardized clinical assessment tool of pathologic and prosthetic hand function: Normative data, reliability, and validity. *Archives of Physical Medicine and Rehabilitation, 83*(6), 776-783.

Malone, J. M., Fleming, L. L., Roberson, J., Whitesides, T. E.,Jr, Leal, J. M., Poole, J. U., & Grodin, R. S. (1984). Immediate, early, and late postsurgical management of upper-limb amputation. *Journal of Rehabilitation Research and Development, 21*(1), 33-41.

Mier, H. I., & Petersen, S. E. (2006). Intermanual transfer effects in sequential tactuomotor learning: Evidence for effector independent coding. *Neuropsychologia, 44*(1), 939-949.

Nagel, M. J., & Rice, M. S. (2001). Cross-transfer effects in the upper extremity during an occupationally embedded exercise. *The American Journal of Occupational Therapy.: Official Publication of the American Occupational Therapy Association, 55*(3), 317-323.

Oldfield, R. C. (1971). The assessment and analysis of handedness: The edinburgh inventory. *Neuropsychologia, 9*(1), 97-113.

Park, J. H., & Shea, C. H. (2002). Effector independence. *Journal of Motor Behavior, 34*(3), 253-270.

Pereira, E. A., Raja, K., & Gangavalli, R. (2011). Effect of training on interlimb transfer of dexterity skills in healthy adults. *American Journal of Physical Medicine &*

Verwijderd: 1
Verwijderd: 22
Verwijderd: 1

Rehabilitation / Association of Academic Physiatrists, 90(1), 25-34. doi: 10.1097/PHM.0b013e3181fc7f6f

Romkema, S., Bongers, R. M., & van der Sluis, C. K. (2013). Intermanual transfer in training with an upper-limb myoelectric prosthesis simulator: A mechanistic, randomized, pretest-posttest study. *Physical Therapy, 93*(1), 22-31. doi: 10.2522/ptj.20120058; 10.2522/ptj.20120058

Schmidt, R. A., & Lee, T. D. (Eds.). (2005). *Motor control and learning: A behavioral emphasis* (4th ed.). Champaign, United States of America: Human Kinetics.

Shea, C. H., Lai, Q., Black, C., & Park, J. H. (2000). Spacing practice sessions across days benefits the learning of motor skills. *Human Movement Science, 19*, 737-760.

Siengsukon, C. F., & Boyd, L. A. (2009). Does sleep promote motor learning? implications for physical rehabilitation. *Physical Therapy, 89*(4), 370-383.

Teixeira, L. A. (2000). Timing and force components in bilateral transfer of learning. *Brain and Cognition, 44*(3), 455-469. doi: 10.1006/brcg.1999.1205

van Lunteren, A., van Lunteren-Gerritsen, G. H., Stassen, H. G., & Zuithoff, M. J. (1983). A field evaluation of arm prostheses for unilateral amputees. *Prosthetics and Orthotics International, 7*(3), 141-151.

Weeks, D. L., Wallace, S. A., & Anderson, D. I. (2003). Training with an upper-limb prosthetic simulator to enhance transfer of skill across limbs. *Archives of Physical Medicine and Rehabilitation, 84*(3), 437-443. doi: 10.1053/apmr.2003.50014

Verwijderd: 1
Verwijderd: 22
Verwijderd: 1

**Pagina 4: [1] Met opmaak**	**romkemas**	**4/29/2013 3:35:00 PM**

Engels (V.S.)

**Pagina 4: [2] Wijzigen**	**Unknown**	

Gewijzigde veldcode

**Pagina 4: [3] Met opmaak**	**romkemas**	**4/29/2013 3:35:00 PM**

Engels (V.S.)

**Pagina 4: [4] Wijzigen**	**Unknown**	

Gewijzigde veldcode

**Pagina 4: [5] Met opmaak**	**romkemas**	**4/29/2013 3:35:00 PM**

Engels (V.S.)

**Pagina 4: [5] Met opmaak**	**romkemas**	**4/29/2013 3:35:00 PM**

Engels (V.S.)

**Pagina 4: [6] Wijzigen**	**Unknown**	

Gewijzigde veldcode

**Pagina 4: [7] Met opmaak**	**romkemas**	**4/29/2013 3:35:00 PM**

Engels (V.S.)

**Pagina 4: [8] Wijzigen**	**Unknown**	

Gewijzigde veldcode

**Pagina 4: [9] Met opmaak**	**romkemas**	**4/29/2013 3:35:00 PM**

Engels (V.S.)

**Pagina 4: [10] Wijzigen**	**Unknown**	

Gewijzigde veldcode

**Pagina 4: [11] Met opmaak**	**romkemas**	**4/29/2013 3:35:00 PM**

Engels (V.S.)

**Pagina 4: [12] Wijzigen**	**Unknown**	

Gewijzigde veldcode

Pagina 4: [13] Met opmaak	romkemas	4/29/2013 3:35:00 PM

Engels (V.S.)

Pagina 4: [14] Wijzigen	Unknown	

Gewijzigde veldcode

Pagina 4: [15] Met opmaak	romkemas	4/29/2013 3:35:00 PM

Engels (V.S.)

Pagina 4: [16] Wijzigen	Unknown	

Gewijzigde veldcode

Pagina 4: [17] Met opmaak	romkemas	4/29/2013 3:35:00 PM

Engels (V.S.)

Pagina 4: [18] Wijzigen	Unknown	

Gewijzigde veldcode

Pagina 4: [19] Met opmaak	romkemas	4/29/2013 3:35:00 PM

Engels (V.S.)

Pagina 4: [20] Wijzigen	Unknown	

Gewijzigde veldcode

Pagina 4: [21] Met opmaak	romkemas	4/29/2013 3:35:00 PM

Engels (V.S.)

Pagina 4: [22] Wijzigen	Unknown	

Gewijzigde veldcode

Pagina 4: [23] Met opmaak	romkemas	4/29/2013 3:35:00 PM

Engels (V.S.)

Pagina 4: [24] Wijzigen	Unknown	

Gewijzigde veldcode

Pagina 4: [25] Met opmaak	romkemas	4/29/2013 3:35:00 PM

Engels (V.S.)

Pagina 4: [26] Wijzigen	Unknown	

Gewijzigde veldcode

Pagina 4: [27] Met opmaak	romkemas	4/29/2013 3:35:00 PM

Engels (V.S.)

Pagina 4: [28] Wijzigen	Unknown	

Gewijzigde veldcode

Pagina 4: [29] Met opmaak	romkemas	4/29/2013 3:35:00 PM

Engels (V.S.)

Pagina 4: [30] Wijzigen	Unknown	

Gewijzigde veldcode

Pagina 4: [31] Met opmaak	romkemas	4/29/2013 3:35:00 PM

Engels (V.S.)

Pagina 4: [32] Wijzigen	Unknown	

Gewijzigde veldcode

Pagina 4: [33] Met opmaak	romkemas	4/29/2013 3:35:00 PM

Engels (V.S.)

Pagina 4: [34] Wijzigen	Unknown	

Gewijzigde veldcode

Pagina 4: [35] Met opmaak	romkemas	4/29/2013 3:35:00 PM

Engels (V.S.)

Pagina 4: [36] Wijzigen	Unknown	

Gewijzigde veldcode

Pagina 4: [37] Met opmaak	romkemas	4/29/2013 3:35:00 PM

Engels (V.S.)

Pagina 4: [38] Wijzigen	Unknown	

Gewijzigde veldcode

Pagina 4: [39] Met opmaak	romkemas	4/29/2013 3:35:00 PM

Engels (V.S.)

Pagina 4: [40] Wijzigen	Unknown	

Gewijzigde veldcode

Pagina 4: [41] Met opmaak	romkemas	4/29/2013 3:35:00 PM

Engels (V.S.)

Pagina 4: [42] Wijzigen	Unknown	

Gewijzigde veldcode

Pagina 4: [43] Met opmaak	romkemas	4/29/2013 3:35:00 PM

Engels (V.S.)

Pagina 4: [44] Wijzigen	Unknown	

Gewijzigde veldcode

Pagina 4: [45] Met opmaak	romkemas	4/29/2013 3:35:00 PM

Engels (V.S.)

Pagina 4: [46] Wijzigen	Unknown	

Gewijzigde veldcode

Pagina 4: [47] Met opmaak	romkemas	4/29/2013 3:35:00 PM

Engels (V.S.)

Pagina 4: [48] Wijzigen	Unknown	

Gewijzigde veldcode

Pagina 4: [49] Met opmaak	romkemas	4/29/2013 3:35:00 PM

Engels (V.S.)

Pagina 4: [50] Wijzigen	Unknown	

Gewijzigde veldcode

Pagina 4: [51] Met opmaak	romkemas	4/29/2013 3:35:00 PM

Engels (V.S.)

Pagina 4: [52] Wijzigen	Unknown	

Gewijzigde veldcode

Pagina 4: [53] Met opmaak	romkemas	4/29/2013 3:35:00 PM

Engels (V.S.)

Pagina 4: [54] Wijzigen	Unknown	

Gewijzigde veldcode

Pagina 4: [55] Met opmaak	romkemas	4/29/2013 3:35:00 PM

Engels (V.S.)

Pagina 4: [56] Wijzigen	Unknown	

Gewijzigde veldcode

Pagina 4: [57] Met opmaak	romkemas	4/29/2013 3:35:00 PM

Engels (V.S.)

Pagina 4: [58] Wijzigen	Unknown	

Gewijzigde veldcode

Pagina 4: [59] Met opmaak	romkemas	4/29/2013 3:35:00 PM

Engels (V.S.)

Pagina 4: [60] Wijzigen	Unknown	

Gewijzigde veldcode

Pagina 4: [61] Met opmaak	romkemas	4/29/2013 3:35:00 PM

Engels (V.S.)

Pagina 4: [62] Wijzigen	Unknown	

Gewijzigde veldcode

Pagina 4: [63] Met opmaak	romkemas	4/29/2013 3:35:00 PM

Engels (V.S.)

Pagina 4: [64] Wijzigen	Unknown	

Gewijzigde veldcode

Pagina 4: [65] Met opmaak	romkemas	4/29/2013 3:35:00 PM

Engels (V.S.)

Pagina 4: [66] Wijzigen	Unknown	

Gewijzigde veldcode

Pagina 4: [67] Met opmaak	romkemas	4/29/2013 3:35:00 PM

Engels (V.S.)

Pagina 4: [68] Wijzigen	Unknown	

Gewijzigde veldcode

Pagina 1: [69] Verwijderd	romkemas	5/3/2013 9:42:00 AM

1

Pagina 1: [69] Verwijderd	romkemas	5/3/2013 9:42:00 AM

22

Pagina 1: [69] Verwijderd	romkemas	5/3/2013 9:42:00 AM

1

Pagina 5: [70] Verwijderd	romkemas	4/23/2013 2:29:00 PM

1. INTRODUCTION AND RATIONALE 7
2. OBJECTIVES 9
3. STUDY DESIGN 9
4. STUDY POPULATION 12
4.1 Population (base) 12
4.2 Inclusion criteria 12
4.3 Exclusion criteria 12
4.4 Sample size calculation 12
5. TREATMENT OF SUBJECTS 12
5.1 Investigational product/treatment 12
5.2 Use of co-intervention (if applicable) 12
5.3 Escape medication (if applicable) 12

6. INVESTIGATIONAL MEDICINAL PRODUCT 12
7. NON-INVESTIGATIONAL PRODUCT 13
8. METHODS 13
8.1 Study parameters/endpoints 13
8.1.1 Main study parameter/endpoint 13
8.1.2 Secondary study parameters/endpoints (if applicable) 13
8.1.3 Other study parameters (if applicable) 13
8.2 Randomisation, blinding and treatment allocation 13
8.3 Study procedures 13
8.4 Withdrawal of individual subjects 18
8.4.1 Specific criteria for withdrawal (if applicable) 18
8.5 Replacement of individual subjects after withdrawal 18
8.6 Follow-up of subjects withdrawn from treatment 18
8.7 Premature termination of the study 18
9. SAFETY REPORTING 19
9.1 Section 10 WMO event 19
9.2 AEs, SAEs and SUSARs 19
9.3 Annual safety report 19
9.4 Follow-up of adverse events 19
9.5 Data Safety Monitoring Board (DSMB)/Safety Committee 19
10. STATISTICAL ANALYSIS 19
10.1 Primary study parameter(s) 19
10.2 Secondary study parameter(s) 19
10.3 Other study parameters 19
10.4 Interim analysis (if applicable) 19
11. ETHICAL CONSIDERATIONS 19
11.1 Regulation statement 19
11.2 Recruitment and consent 20
11.3 Objection by minors or incapacitated subjects (if applicable) 20
11.4 Benefits and risks assessment, group relatedness 20
11.5 Compensation for injury 20
11.6 Incentives (if applicable) 20
12. ADMINISTRATIVE ASPECTS, MONITORING AND PUBLICATION 20
12.1 Handling and storage of data and documents 20
12.2 Monitoring and Quality Assurance 20
12.3 Amendments 20
12.4 Annual progress report 21
12.5 End of study report 21
12.6 Public disclosure and publication policy 21
13. STRUCTURED RISK ANALYSIS 21

13.1 Potential issues of concern 21
13.2 Synthesis 21

Pagina 9: [71] Verwijderd **romkemas** **3/12/2013 11:22:00 AM**

Healthy adults										
Training	**Functional tasks**		**Force control tasks**		**Reaching**		**Grasping**		**Control group**	
Participants	*3 men dom; 3 men n-dom, 3 women dom; 3 women n-dom*		*3 men dom; 3 men n-dom, 3 women dom; 3 women n-dom*		*3 men dom; 3 men n-dom, 3 women dom; 3 women n-dom*		*3 men dom; 3 men n-dom, 3 women dom; 3 women n-dom*		*3 men dom; 3 men n-dom, 3 women dom; 3 women n-dom*	
**Day**	**Test**	**Practice**	**Test**	**Practice**	**Test**	**Practice**	**Test**	**Practice**	**Test**	**Practice**
1	Pretest	20 min	Pretest	20 min	Pretest	20 min	Pretest	20 min	Pretest	-
2	-	20 min	-	20 min	-	20 min	-	20 min	-	-
3	-	20 min	-	20 min	-	20 min	-	20 min	-	-
4	-	20 min	-	20 min	-	20 min	-	20 min	-	-
5	Posttest	20 min	Posttest	20 min	Posttest	20 min	Posttest	20 min	Posttest	-
7	Retention test	-	Retention test	-	Retention test	-	Retention test	-	Retention test	-
